# Sequential Modelling of the Effects of Mass Drug Treatments on Anopheline-Mediated Lymphatic Filariasis Infection in Papua New Guinea

**DOI:** 10.1371/journal.pone.0067004

**Published:** 2013-06-24

**Authors:** Brajendra K. Singh, Moses J. Bockarie, Manoj Gambhir, Peter M. Siba, Daniel J. Tisch, James Kazura, Edwin Michael

**Affiliations:** 1 Department of Biological Sciences, University of Notre Dame, Notre Dame, Indiana, United States of America; 2 Liverpool School of Tropical Medicine and Hygiene, University of Liverpool, Liverpool, United Kingdom; 3 Department of Infectious Disease Epidemiology, Imperial College London, London, United Kingdom; 4 Papua New Guinea Institute of Medical Research, Goroka, Eastern Highlands Province, Papua New Guinea; 5 Center for Global Health and Diseases, Case Western Reserve University, Ohio, United States of America; Swiss Tropical & Public Health Institute, Switzerland

## Abstract

**Background:**

Lymphatic filariasis (LF) has been targeted by the WHO for global eradication leading to the implementation of large scale intervention programs based on annual mass drug administrations (MDA) worldwide. Recent work has indicated that locality-specific bio-ecological complexities affecting parasite transmission may complicate the prediction of LF extinction endpoints, casting uncertainty on the achievement of this initiative. One source of difficulty is the limited quantity and quality of data used to parameterize models of parasite transmission, implying the important need to update initially-derived parameter values. Sequential analysis of longitudinal data following annual MDAs will also be important to gaining new understanding of the persistence dynamics of LF. Here, we apply a Bayesian statistical-dynamical modelling framework that enables assimilation of information in human infection data recorded from communities in Papua New Guinea that underwent annual MDAs, into our previously developed model of parasite transmission, in order to examine these questions in LF ecology and control.

**Results:**

Biological parameters underlying transmission obtained by fitting the model to longitudinal data remained stable throughout the study period. This enabled us to reliably reconstruct the observed baseline data in each community. Endpoint estimates also showed little variation. However, the updating procedure showed a shift towards higher and less variable values for worm kill but not for any other drug-related parameters. An intriguing finding is that the stability in key biological parameters could be disrupted by a significant reduction in the vector biting rate prevailing in a locality.

**Conclusions:**

Temporal invariance of biological parameters in the face of intervention perturbations indicates a robust adaptation of LF transmission to local ecological conditions. The results imply that understanding the mechanisms that underlie locally adapted transmission dynamics will be integral to identifying points of system fragility, and thus countermeasures to reliably facilitate LF extinction both locally and globally.

## Introduction

Lymphatic filariasis (LF), a highly debilitating vector-borne macroparasitic disease of humans, has been targeted by the World Health Organization (WHO) for global eradication [1]–[5]. This has led to the rapid development and financing of large scale national-level intervention programs based primarily on annual mass drug administrations (MDA), which has led to impressive reductions in LF infection prevalences in endemic populations globally [6]. However, as infection levels have fallen in endemic communities, questions have been raised regarding the need for improved understanding of the dynamical processes that underlie infection persistence and extinction dynamics, and hence the controllability or eradicability of the disease [7]–[11]. A practical question in this regard revolves around the nature of LF extinction dynamics, including the numerical values of LF transmission/infection endpoints in endemic communities, the resolution of which is key to reliably determining when parasite transmission has been interrupted and interventions can therefore be stopped [12].

Our previous work has highlighted that both inherent complexity in transmission dynamics and parameter uncertainty can confound the prediction of extinction endpoints for complicated parasitic systems, such as LF [7], [9], [11]–[13]. In particular, this work has showed that complex ecological dynamics due to high sensitivity to initial conditions and other locally varying climatic and geographic factors mean that parasite transmission dynamics is likely to be highly variable in space and time [12], with the result that LF infection endpoints or breakpoints (*e.g.* threshold values of the microfilaria (mf) prevalence in humans below which infection cannot sustain itself) may vary considerably from site to site. Although it was shown that between-site heterogeneities in a few biological and socio-ecological parameters controlling the intensity and distribution of worm burdens in the human host population may underlie this variability, the results also demonstrated that a significant source of the variability reflects uncertainties surrounding parameter values of key transmission variables [12]. While a part of this uncertainty reflects epistemic uncertainty regarding transmission heterogeneity, it is clear that a major portion is also due to the limited quantity and quality of site-specific data used to develop/run existing models [12], [14], [15]. This implies that reducing parameter uncertainty will require the need for data-model integration methods that can assimilate information and update initial parameter values as new information regarding LF transmission dynamics becomes available either from different sites or over time from individual sites [7], [9], [11]–[13], [15]–[18].

In the context of the current initiative to eradicate LF, effective model updating using longitudinal infection data from sites undergoing interventions serves another crucial function, viz. providing an important means to investigate possible temporal patterns in the data. A particular focus in this regard of direct import to managing LF eradication is the determination of whether the updating of initial model fits (to baseline infection data) using follow-up data after the start of interventions will result in significantly altered estimates, particularly in the critical biological parameters underlying parasite transmission. If this occurs to the extent of bringing about dynamical behaviour changes, then it may raise the real possibility of the emergence of temporally varying endpoints as parasite interventions proceed. The outcome of such a qualitative change in overall system dynamics will, by affecting attractors, basins of attraction and stability, be that LF endpoints could become dynamic ‘moving’ targets. The implication of such changes for LF elimination may be the prolonging of interventions in some settings, while in others we may need to develop and implement other adaptive control strategies, including supplementation by vector control or with more frequent mass treatments. If on the other hand, model updates show that values of critical biological parameters underpinning transmission do not vary appreciably or are robust in response to the effect of interventions, then it might be possible to derive estimates of transmission endpoints from post-intervention monitoring data. This will be a significant step forward in the management of elimination efforts against LF since it would allow the estimation of target endpoints for interventions even in the absence of baseline infection data for those sites which have only post-intervention monitoring data. Such results would also facilitate uncovering of the environmental or other conditions that constrain or bound the transmission and extinction dynamics of LF to an endemic setting [19], [20]. This will in turn enable insights into countermeasures that can disrupt the local robustness in transmission, increase system fragility and more reliably push the locally adapted transmission system into extinction [19], [21]–[23].

Sequential calibration/updating of LF transmission models using post-intervention follow up data on infection levels could also produce additional information relevant to predicting the impacts of interventions. For example, such model updates will be important for a more direct estimation and evaluation of drug related parameters, such as worm and mf killing efficacies, reduction in worm fecundity, and drug lasting effects that impact the duration of suppression in mf production [10], [24]–[26]. Present estimates of these effects are still largely based on best guestimates [7]; better attempts to quantify such parameters using field data is thus a distinct need and will be critical to improving control predictions and hence intervention planning in endemic localities.

In a similar vein, the sequential fitting of models to follow-up data could additionally offer the potential for allowing the backward estimation of pre-control baseline infection patterns. Reconstructions of historical baseline infection patterns (particularly in terms of age-profiles of mf prevalence levels) will be important because many LF intervention monitoring sites currently do not have such data even though this information is clearly essential for predicting trajectories in infection declines from baseline levels as a result of applied interventions [27], [28], which will be essential for evaluating if interventions are progressing as expected [27], [29].

Here, we report on our efforts to undertake a first systematic examination of each of the above issues in the context of a detailed field trial to evaluate the impact of MDA for reducing LF infection in endemic communities from the Dreikikir region of Papua New Guinea [30]. We begin by first describing the extension and use of a recently developed numerical modelling and Bayesian Melding data-model integration tool to sequentially fit the *Anopheles*-mediated LF transmission model [11] to baseline and follow-up human mf age-prevalence data recorded from each of our Dreikikir study communities. We then use the modelling results to address the following set of specific questions: 1) do biological parameters controlling LF transmission remain stable (with regards to baseline estimates) in the face of the specific interventions implemented in each community; 2) is it possible to use the fits to data from each intervention period to reliably perform backward extrapolation to reconstruct baseline age-infection patterns; 3) is it possible to use the post-intervention infection monitoring data to estimate LF transmission/infection breakpoints consistent with those estimated using baseline only data; and 4) can the modelling and fitting framework developed in this study allow better estimates of drug treatment-related parameters, including determination if such parameters may also vary in their values between treatment populations? We end by discussing the significance of the findings regarding the ecological factors that may underlie LF transmission and extinction dynamics in local settings, the importance of good quality intervention monitoring data, including drug coverage information, and the value of the applying the Bayesian model-data assimilation method used here, for guiding the WHO-directed program to achieve the eradication of LF globally.

## Materials and Methods

### Data

The data used in this analysis represent baseline and annual follow-up monitoring data on changes in lymphatic filariasis infection prevalences obtained from five communities (Peneng, Albulum, Yauatong, Nanaha and Ngahmbule) in the Dreikikir region of Papua New Guinea (PNG) [30]–[34]. These data were collected as part of an open-label field study to compare the population impacts of a single annual mass administration of diethylcarbamazine plus ivermectin (DEC+IVR) with that of a single annual dose of DEC alone for reducing LF infection transmitted by anopheline mosquitoes. The data on individual host mf status for the baseline period was collected in 1994 before the start of mass treatment intervention and thereafter at the end of one-year intervals for a period of 5 years [34]. Three of the five villages (Peneng, Albulum, and Yauatong) were classified as high transmission communities and the remaining as exposed to a moderate rate of transmission based on annual mosquito biting rates [30]–[32]. Annual population sizes, totals of individuals sampled, numbers of mf-positives out of these samples, yearly drug coverages and the drug regimen for each of these villages, are provided in Table 1. Infection status of individuals were assessed using 1 ml of blood, whereas prevailing vector abundances (in terms of annual biting rates per person) were determined using man landing catches in all communities [30], [31]. Note that as per WHO guidelines at the time of the field trials, individuals under five years of age were excluded from treatment with drugs during the MDA period in any of these villages [30].

**Table 1:** Annual survey data for lymphatic filariasis (LF) mf prevalence for each of the five PNG study villages used in this work.

	High Transmission Zone									Low Transmission Zone					
Village	Peneng			Albulum			Yauatong			Nanaha			Ngahmbule		
Regimen	DEC+IVR			DEC+IVR			DEC alone			DEC+IVR			DEC alone		
ABR	8194			42328			37052			11611			4346		
Year	T (PS)	Mf+	C	T (PS)	Mf+	C	T (PS)	Mf+	C	T (PS)	Mf+	C	T (PS)	Mf+	C
1993–94	63(69)	42	n/a	50(71)	40	n/a	131(169)	121	n/a	211(281)	116	n/a	346(428)	177	n/a
1994–95	65(67)	40	50.38	60(60)	44	61.54	144(145)	104	66.19	238(247)	115	71.60	343(353)	118	70.24
1995–96	88(89)	18	77.98	69(69)	26	63.46	111(113)	58	57.75	208(210)	57	65.80	299(308)	79	60.69
1996–97	89(89)	12	75.21	70(70)	18	66.35	123(123)	44	62.57	196(211)	26	62.82	318(323)	30	65.20
1997–98	92(92)	5	68.42	75(75)	11	58.20	138(138)	27	65.85	221(224)	2	66.77	235(236)	11	47.44
1998–99	109(109)	4	71.92	64(64)	3	52.89	91(91)	8	43.69	166(172)	1	49.40	290(294)	5	56.40

Table keys: ABR - Annual Biting Rate (average number of mosquito bites per person per year); **T** - the total number of individuals sampled, with the bracketed numbers showing the total population sizes (**PS**) of the study villages; **Mf+** - the number of mf-positive samples out of the total individuals sampled; **C** - population-level drug coverage, the percentage (%) of people who took the prescribed drug regimen during annual mass drug treatments; **DEC** - diethylcarbamazine; **IVR** – ivermectin. The baseline survey was done in 1993–94. The 5-year intervention period ranged from 1994–95 through 1998–99. Wherever required in the paper, the post-intervention years are indicated by Year 1 (*i.e.*, 1994–95) through Year 5 (*i.e.*, 1998–99).

### Methods

#### Deterministic model of anopheline-mediated LF transmission

We employed the recently developed *Anopheles*-vectored transmission model of LF to carry out the modelling work in this study [7], [9], [11], [12]. Briefly, the state variables of this hybrid coupled partial differential and differential equation model vary over age (*a*) and/or time (*t*), representing changes in the adult worm burden per human host 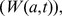 the mf level in the human host modified to reflect infection detection in a 1 ml blood sample 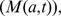 the average number of infective L3 larval stages per mosquito (*L*), and a measure of immunity 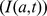 developed by human hosts against L3 larvae. The state equations comprising this model are:
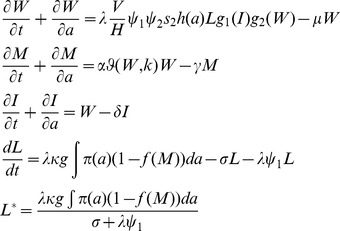



The above equations involve partial derivatives of three state variables (*W* - worm load; *M* - microfilaria intensity; *I* - immunity to acquiring new infection due to the pre-existing worm load), whereas given the faster time scale of infection dynamics in the vector compared to the human host, the infective L3-stage larval density in mosquito population is modeled by an ordinary differential equation. This basic coupled immigration-death structure of the model, as well as our recent extensions to reflect transmission by anopheline mosquitoes in endemic areas, such as in the Dreikikir region in PNG, have been extensively discussed previously [7], [11], [12], [35], [36]. The parameters and functions of the model and L^*^ are described in **Table 2**.

**Table 2:** Description the basic LF model parameters and functions used in the model.

Parameter	Definition (*units*)	Range	Source
Intrinsic ***Biological*** parameters			
***λ***	Number of bites per mosquito (*per month*)	[5], [15]	[11], [12], [37], [38]
***ψ_1_***	Proportion of L3 leaving mosquito per bite	[0.12, 0.7]	[39]
***ψ_2_s_2_***	The establishment rate^1^	[0.0000398, 0.00364]	[11], [12], [40]
***μ***	The worm mortality rate (*per month*)	[0.008, 0.018]	[11], [12], [41]–[44]
***α***	Production rate of microfilariae per worm (*per month*)	[0.25, 1.5]	[11], [12], [39]
***γ***	The death rate of the microfilariae (*per month*)	[0.08, 0.12]	[12], [39], [43]
***g***	Proportion of mosquitoes which pick up infection when biting an infected host	[0.259, 0.481]	[12], [45]
***σ***	Death rate of mosquitoes (*per month*)	[1.5, 8.5]	[12], [40]
***κ***	Maximum level of L3 given mf density	[3.955, 4.83]	[12]
***c***	Strength of acquired immunity	[0.0000003, 0.0109]	[12]
*δ*	Immunity waning rate (*per month*)	[0, 0.000001]	[12]
Extrinsic ***Biological*** parameters			
***V/H***	Ratio of number of vector to hosts	*MBR*/***λ***	[12], data
***H_lin_***	A threshold value used in *h*(*a*) to adjust the rate at which individuals of age *a* arebitten: linear rise from 0 at age zero to 1 at age *H_lin_* in months (*h*(*a*) = *a*/*H_lin_* for *a*<*H_lin_*;*h*(*a*) = 1 otherwise)	[12, 240] months	[12], [36]
***r***	Gradient of mf uptake^2^	[0.0495, 0.22]	[12]
***I_C_***	Strength of immunosuppression^3^	[0.5, 5.5]	[12]
***S_C_***	Slope of immunosuppression function^4^ (*per worm/month*)	[0.01, 0.19]	[12]
***k_0_***	The basic location parameter of negative binomial distribution used in aggregationparameter (*k* = *k_0_*+*k_Lin_M*)	[0.000036, 0.00077]	[12], [46], [47]
***k_Lin_***	The linear rate of increase in the aggregation parameter defined above	[0.00000024, 0.282]	[12], [46], [47]
***Drug’s efficacy related parameters & Coverage***			
***ω***	Worm killing efficacy (instantaneous)	[0.1, 0.85]	[7]
***ε***	Microfilariae killing efficacy (instantaneous)	[0.55, 0.95]	[7]
***δ*** *_reduc_*	Reduction in the worm’s fecundity over a period of time ***P***	[0.55, 0.95]	[7]
***P***	A time period during which the drug remains efficacious in reducing the fecundityof the surviving adult worms	[1], [6]	[7]
***C***	Percentage of the populations of the study villages administered the drug	[43.69, 77.98]	data
***Description of the functions used in the model***			
**Function**	**Definition** 5	**Parameters**	**Source**
*π(a)*	Probability that an individual is of age *a*	Human age *a* in month	[12], [36]
*φ(W,k)*	Adult worm mating probability	*k* – negative binomial aggregation parameter	[11], [12], [48]
*g_1_(I)*	Immunity to larval establishment	*c* – strength of immunity to larval establishment	[12]
*g_2_(W)*	Host immunosuppression	*I_C_* – strength ofimmunosuppression; *S_C_* – slope ofimmunosuppression	[12]
*f(M)*	Population-averaged vector uptake function	*κ* – maximum level of L3 given mf intensity; *r* – gradient of mf uptake	[11], [12]

1 The proportion of L3-stage larvae infecting human hosts that survive to develop into adult worms [12].

2 The gradient of mf uptake *r* is a measure of the initial increase in the infective L3 larvae uptake by vector as *M* increases from 0 [12], [36].

3 The facilitated establishment rate of adult worms due to parasite-induced immunosuppression in a heavily infected human host.

4 The initial rate of increase by which the strength of immunosuppression is achieved as *W* increases from 0 [8].

5 Mathematical expressions of the 5 functions:

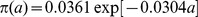
; 
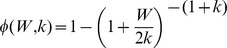
; 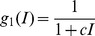; 
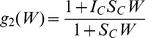
; 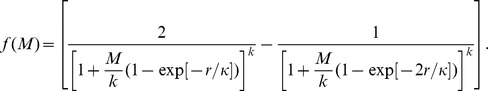

All the intrinsic and extrinsic biological parameters, except *V/H*, described in this table are estimated. The *V/H* is adjusted in way that 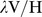 returns the value of monthly biting rate (MBR). The four drug-related parameters are also estimated. The annually observed population-level MDA coverage *C*, on which information is available, is used as an input during the model runs for the mass drug intervention. The functions, except 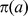, described above contain one or two model parameters as well as one of the state variables (*W*, *M*, *I*) describing infection in humans. The L^*^ represents the average number of infective L3 larval stages per mosquito at the endemic situation.

Note that the biological parameters of the model are classified into site-specific and site-invariant parameters, based on results from our previous work which suggest that it is useful to distinguish between biological parameters specific to the disease, and which therefore can be assumed to apply to parasite and vector populations that range over extended geographical areas, and those which vary between localities [12], [49].

### The Bayesian Melding Framework

Our strategy was essentially to integrate follow-up field observations on LF infection in populations undergoing mass drug treatments with simulation model outputs to, firstly, update the uncertainty in model parameters, and then use the calibrated model to examine: (1) the stability of transmission-driving parameters in the face of mass treatments, and (2) the utility of sequential model fits to allow reliable back-extrapolation of baseline infection dynamics as well as estimation of infection endpoints and drug treatment-related parameters. We used the statistical framework founded on the Bayesian Melding (BM) algorithm to address this sequential model-fitting and analysis problem [50]–[55].

The BM approach may be described as a procedure whereby two *priors* on a model output are compared and “melded” together in order to obtain the posterior parameter space in which the model may reliably explain the underlying natural system dynamics [52], [56]. One of the priors on model output is the set of observed data; for example, in our case the survey data on LF prevalence collected from each endemic community before the start and during the implementation of a mass drug treatment program. The other output prior is the model generated values of the state variables, such as *W* or *M*. Thus, the BM procedure is a method for reconciling several sources of prior information (on both input parameters and on model outcomes relative to data) to constrain the acceptable solution space of the input parameters. In the form of the method we implemented here, we initially assigned vague or uniform prior distributions for each of the model input parameters (except for the mosquito biting rate, which was fixed to the values of the monthly biting rate (MBR; see Table 2) measured at baseline in the study data) to reflect our initial incomplete knowledge regarding their values, while for assessing reasonableness of model outputs to data, a binomial likelihood function was constructed to capture the distribution of the observed age-mf prevalence data:
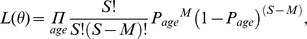
where *M* is the total microfilaraemic (i.e. mf positives) samples out of the total *S* blood samples collected from people in the *age* ^th^ age-group, and the term 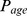 is the modelled mf prevalence in the same age-group. The 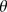 represents a set of the model parameters, termed as parameter vector.

Following the methodological protocol presented in our earlier work [11], [12], we then realized the multidimensional space defined by the set of prior distributions by randomly sampling *N* parameter vectors from their defined ranges. This procedure is summarized as follows. There are 18 parameters in the model (Table 2), which together form a parameter vector, *θ_i_*. Let the i-*th* element of a single parameter vector *θ_i_* be defined by *θ_ii_*, ie, 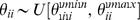, where 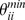 and 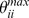 are, respectively, the minimum and maximum values of that element parameter 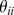. With each of these parameter vectors, , the model is simulated and the posterior distribution of model parameters 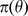 obtained by fitting the model to observed data. We then used the sampling-importance-resampling (SIR) algorithm to resample, with replacement, from the above model parameter sets with the probability of acceptance of each resample 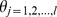 probable to its weight 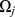, which is proportional to the corresponding likelihood 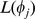 for the data, *i.e.* 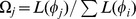. A typical value of *l* for the results presented in this paper was around 500, and these parameter sets are then used to generate distributions of variables of interest from the model (*e.g.* age-prevalence curves, worm breakpoints, drug-related parameter values and post-treatment infection trajectories), including measures of their uncertainties [12].

### Sequential BM Model Fitting to LF Infection Data Following Drug Interventions

The sequential BM updating approach involves three steps. First, we fitted the model using BM to the age-mf prevalence baseline data from each community to obtain the first pre-control posterior distributions of the input parameters for each locality, say π*_1_*(Π), from the SIR, as discussed above. Second, we use the model thus parameterized together with a set of drug-related model parameter vectors (see below) to predict the state of age-mf infection one year after the first post annual treatment. Third, we then update the biological model parameters by applying the SIR procedure to the first post annual treatment field age-infection data to get a new postmodel distribution, π_2_(Π), using as prior inputs the posterior distributions obtained from the baseline fit. This conditional approach thus sequentially filters the parameter values over time with new data thereby reducing parameter uncertainty [57]. This procedure was then applied iteratively, adding further new post-intervention field data over time, in order to progressively filter and thus update the initially obtained parameter estimates [52], [57].

### Modelling the Effects of Annual Mass Drug Administration

The impact of annual mass drug treatment was modelled by assuming that anti-filarial treatment with the currently used drug regimens acts by killing certain fractions of the populations of adult worms and mf instantly after drug administration [7]. Assume these fractions to be *ω* for adult worms, and *ε* for mf. These killing effects are easily incorporated into the basic model and the population sizes of worms and microfilariae are calculated after drug treatment by modifying the populations of each parasite stage obtained immediately prior to the treatment by:
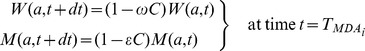
where 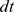 is a short time-period since the time-point 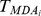 when the i-*th* MDA was administered. During this short time-interval, a given proportion of adult worms and mf (as specified by values of the drug efficacy rates for these life stages, 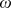 and 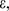 respectively) are instantaneously killed. The parameter *C* is the population-level drug coverage.

Apart from instantaneous killing of adult worms and mf, filariasis drug regimens are also thought to reduce the production of mf by worms surviving each MDA. Here we modeled this effect by introducing a new parameter (denoted by *δ_reduc_*) as follows:
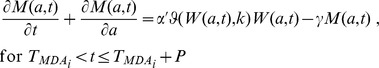
where 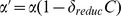 reflects the suppressed fecundity (over a period of *P* months since the i-*th* MDA) of adult worms that survive the administration of drugs at each MDA.

The four drug-related parameters (*ω*, *ε*, *δ_reduc_, and P*) together with their range of non-informative prior distributions are listed in Table 2. The first MDA round is implemented in the model immediately after the baseline survey data is fitted, with the remaining four MDA effects implemented annually thereafter. Note that site-specific annual drug coverages are known for each locality/field data set and are therefore not treated as a variable/estimable element of the drug-related parameter vector.

### Stability of Transmission Parameters and Backwards Extrapolation of Baseline Community Infection Age-patterns

This was carried out by fitting the dynamic model using the sequential BM approach described above to the annual field age-mf infection data obtained from each community following the 1^st^ through to the 5^th^ year of mass drug treatments, and then running the model for each of the *l* best non-drug related parameter vectors in order to derive the mf age-prevalence equilibrium/endemic states to be expected at baseline. The temporal stability of the distributions of transmission parameters obtained at each time period was determined using the univariate Kolmogorov-Smirnov (KS) test [58], whereas we evaluated the goodness of fit of each back calculated model prediction to observed baseline age-infection data from each community, by calculating the so-called Monte Carlo *p*-value using a modified version of the Pearson’s χ^2^ goodness-of-fit test described in [59]. Both age-stratified as well as age-aggregated Monte Carlo *p*-values were determined in order to evaluate the quality of model fits.

### Calculation of Worm Breakpoints

We applied a numerical stability analysis approach based on varying initial values of L^*^ (see details of the procedure provided in [11]), to each of the SIR selected model vectors in order to calculate the distribution of mf prevalence breakpoints and threshold biting rates (TBR) expected in each study community. There are two essential steps in this approach. In the first step, we progressively decrease *V/H* from its original value to a threshold value *below* which the model always converges to zero mf prevalence, regardless of the values of the endemic infective larval density L^*^. The product of 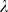 and this newly found *V/H* value is termed as the threshold biting rate (TBR). Once the threshold biting rate is discovered, the model at TBR can settle to either a zero (trivial attractor) or non-zero mf prevalence depending on the starting value of L^*^. Therefore, in the next step, while keeping all the model parameters unchanged, including the new V/H, and by starting with a very low value of L^*^ and progressively increasing it in very small step-sizes we estimate the minimum L^*^ below which the model predicts zero mf prevalence and above which the system progresses to a positive endemic infection state. The corresponding mf prevalence at this new L^*^ value is termed as the worm breakpoint [11]. We compared the worm breakpoint values obtained from sequential BM fits of the model to longitudinal infection data during each year of MDA with those estimated via direct fits to baseline age-mf infection data both formally and also using quadratic discriminant analysis (the klaR package in R), to determine if the sequential estimates are consistent with those quantified directly from baseline data in each study village. As above, the univariate KS test was used to formally test for differences between the baseline and sequential estimates in each case.

## Results

### Model Fits to Annual Intervention Data

**Figure 1:**
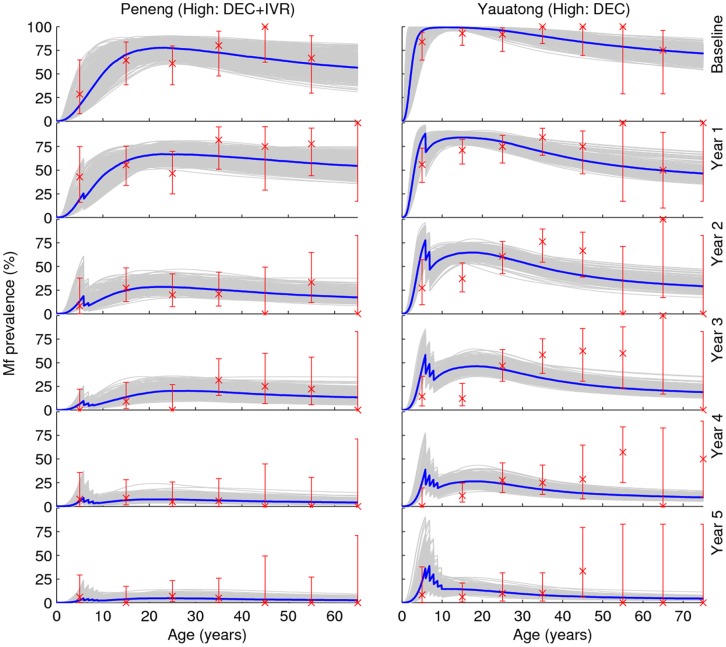
Predicted age-profiles of mf-prevalence from model fits to observed baseline and longitudinal post-intervention infection data. Model outputs and observed data are shown for two of the PNG study villages exposed to high LF transmission intensity, and for both drug regimens used: Peneng (DEC+IVR) and Yauatong (DEC alone). Individual 500 best-fit model simulations are shown in grey while the thick blue line represents the median value of these curves. The observed data points (*crosses*) with 95% binomial credible intervals are shown at the mid-points of each 10-yearly age-group. The results for the remaining three study villages are exactly similar (**Figure S1** in Supporting Information).

**Figure 2:**
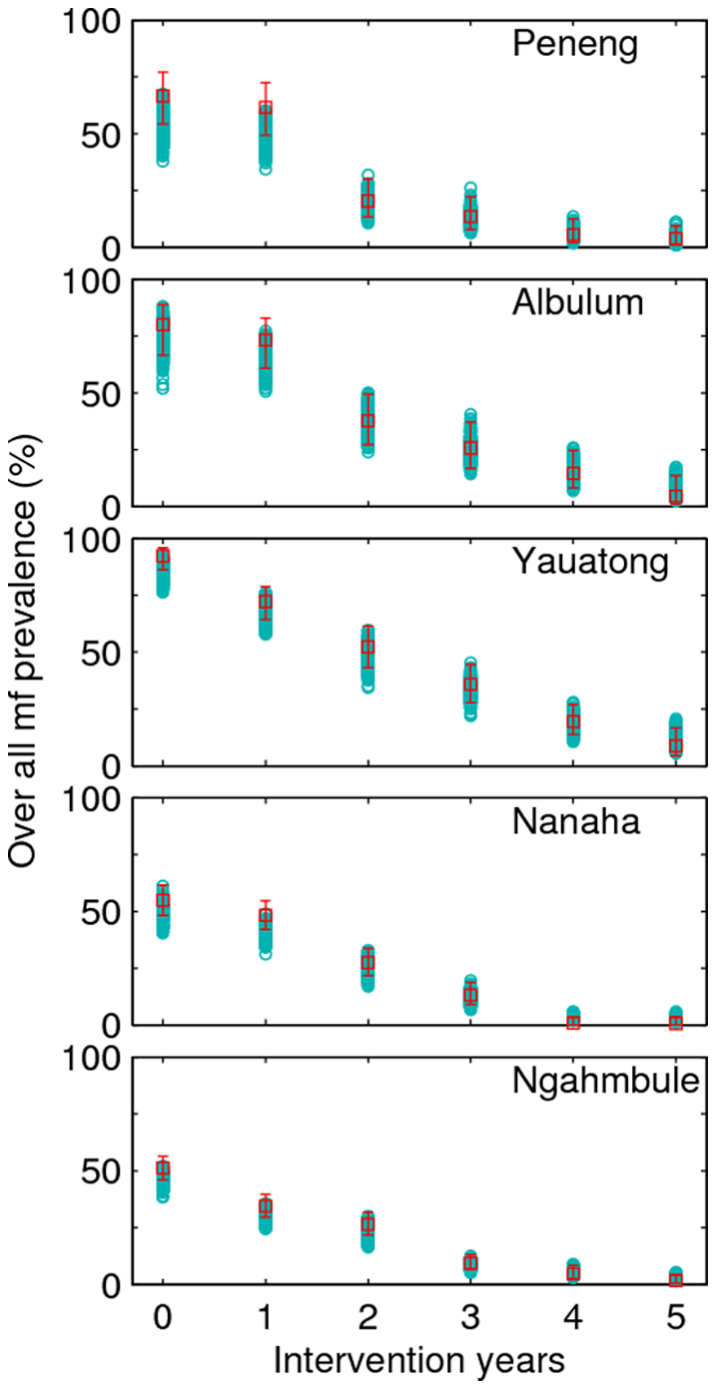
Comparison of the trends in overall community-level mf prevalence predicted by the model with the observed baseline and post-intervention infection data collected longitudinally during the mass treatment programme. Model outputs and observed infection data are shown for all the five PNG study villages. The observed overall community prevalence collected at a yearly interval from each study site are depicted by empty red *squares* together with their corresponding 95% binomial credible intervals (bars). The baseline data (at the 0^th^ year in 1993–94) were collected soon before the start of the first mass drug administration round, and the five post-intervention mf prevalence data were collected annually a year after each mass drug treatment from the first through the 5^th^ MDA round (see **Table 1**). Note that there are 500 modelled data points (shown by empty cyan *circles*) from the 500 best fits.

The age-profiles of mf-prevalence generated by the Anopheline LF model fits (grey curves) to the baseline and five annual post-intervention data from two of the high transmission study villages given either the DEC+IVR regimen (Peneng) or DEC alone (Yauatong) are shown in **Figure 1**. The corresponding model fits to the baseline and intervention data from the remaining three PNG villages are shown in **Figure S1** in Supporting Information S1. **Figure 2** shows the overall mf prevalences predicted by the model in comparison with the longitudinally observed baseline and post-intervention overall community mf prevalences recorded for each of the five study villages. Together, these results show clearly that the BM-based model-data assimilation method developed in this study is capable of reproducing the temporal changes in overall, and age-stratified, prevalences in mf, arising from the implemented mass drug treatments consistent with observed data in each of the study communities (Monte Carlo *p*-values >0.05 in each case (**Table S1** in Supporting Information S1)), although as expected the fits to mf-age-prevalences are comparatively better for the study villages with the lowest variability in this infection measure, viz. Nanaha and Ngahmbule (**Figure S1** in Supporting Information S1) owing primarily to their bigger sample sizes (**Table 1**).

### Stability of Model Parameters

**Figure 3:**
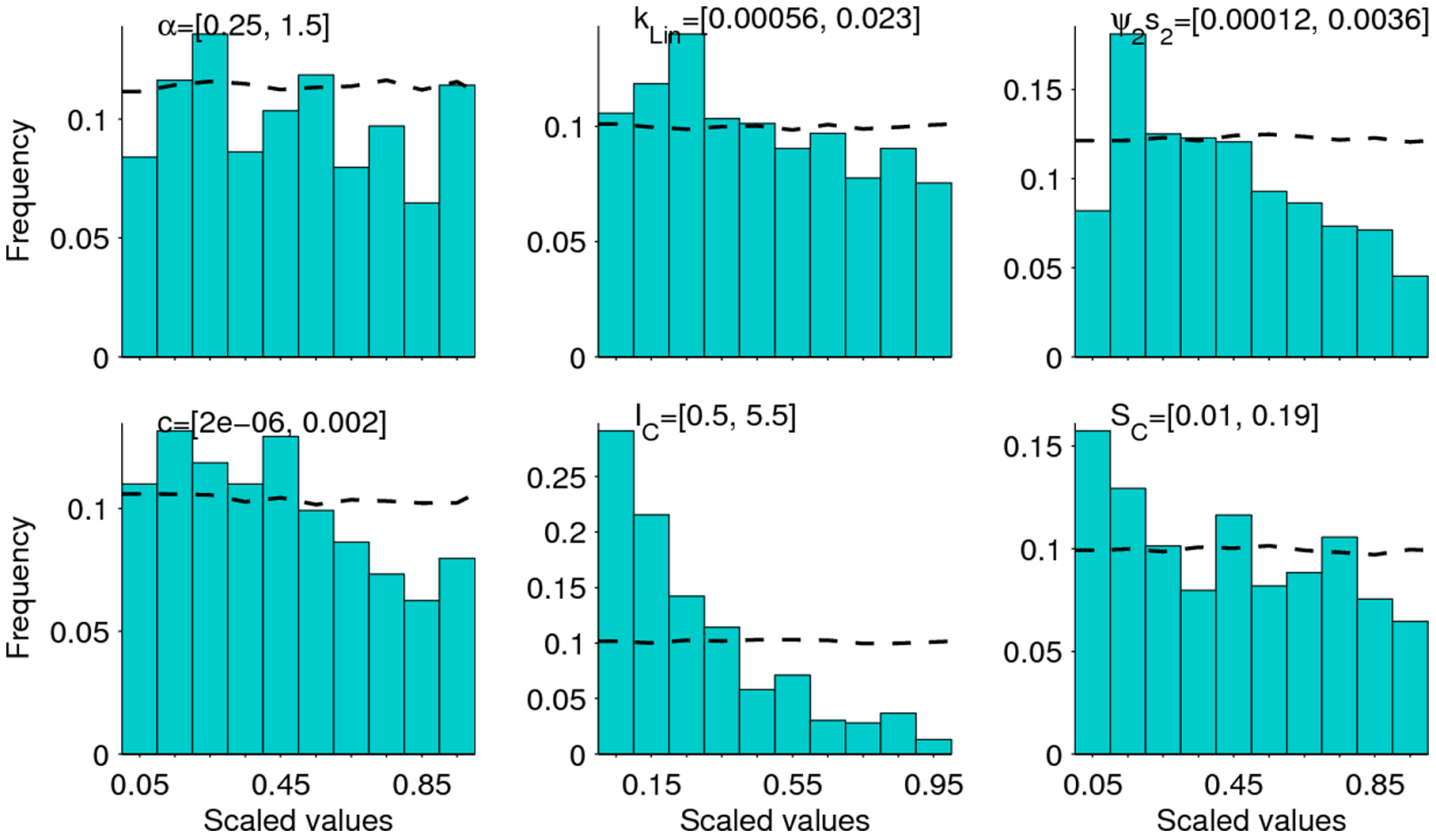
Posterior distributions of model parameters. The results are shown for 6 model parameters, *α, k_Lin_*, *ψ_2_s_2_*, *c*, *I_C_* and *S_C_*, whose posteriors changed significantly and consistently from their initially assigned uniform priors when the LF model was fitted to the baseline data in each study village. The results in the figure illustrate the estimated posterior parameter shifts for the village of Peneng. Dashed lines show the flat non-informative priors, while the bars denote the frequency distributions of the posterior values for each parameter. The parameter values along the x-axis are normalized for comparative purposes. The figures in square brackets at the top of each plot depict the actual range of the posterior values estimated for each parameter.

**Figure 4:**
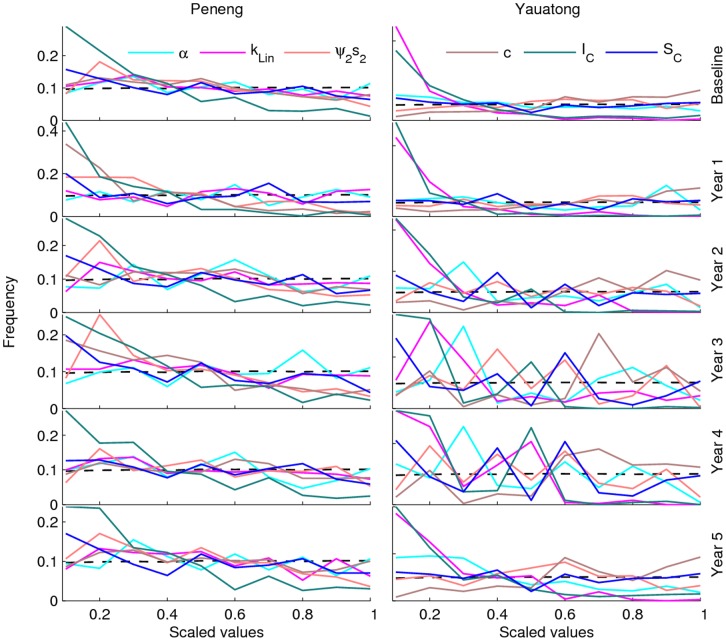
Stability in model parameters during intervention period. Comparison of changes in the posteriors of the parameters *α, k_Lin_*, *ψ_2_s_2_*, *c*, *I_C_* and *S_C_* obtained via sequential model fitting to annual infection data recorded over the 5-year intervention period in the high transmission villages of Peneng and Yauatong. Solid coloured lines denote of the relative frequencies of the posterior distributions of each of the six model parameters. The x-axis shows the scaled values of the posterior distributions for each parameter. The dashed line shows the initially assigned flat non-informative priors.

As in our previous work [12], the results show that the Bayesian updating procedure used here when applied to baseline age-mf prevalence data can effectively refine initially assigned model parameter values. As indicated in **Table S2** in Supporting Information S1, almost all the values initially assigned to the present model parameters were updated by the procedure, although as also noted previously [12], an important finding is that this was dependent on study sample size, with increasing changes in the posterior distributions occurring for those study communities (e.g. Nanaha, Ngahmbule) with the larger sample sizes. Across all study sites, however, only six parameters, namely, α, *k_Lin_*, *ψ_2_s_2_*, *c, I_C_* and *S_C_*, which represent the fecundity rate per worm, the linear rate of increase in the aggregation parameter, the parasite establishment rate, the strength of acquired immunity to L3 larvae, the strength of immunosuppression and the slope of immunosuppression function, respectively, were consistently found to have their posterior distributions significantly altered from their assigned non-informative uniform prior distributions when the model was fitted to the baseline data (**Figure 3**). We next simulated the effects of each MDA regime using actually observed community drug coverage rates, and used the fits of these simulations to data from each study village to assess if the values of any of these biological/ecological parameters remained temporally invariant. The results demonstrated that these six parameters remained remarkably fixed throughout the intervention period in all study sites (**Figure 4**), with their posterior distributions retaining the same individual baseline distributional shapes in each case. A more formal test of how stable or robust to perturbations all the model parameters, including these six model parameters, were during the 5-year post-treatment period was carried out using the univariate KS test [58]. This indicated that almost all the biological parameters of the model remained at their initial baseline posterior distributions during the entire 5-year study period when infection in each of the five LF communities were going through their annual intervention-induced perturbations (**Table S2** in Supporting Information S1).

### Estimation of the Drug Efficacy Related Parameters

**Figure 5:**
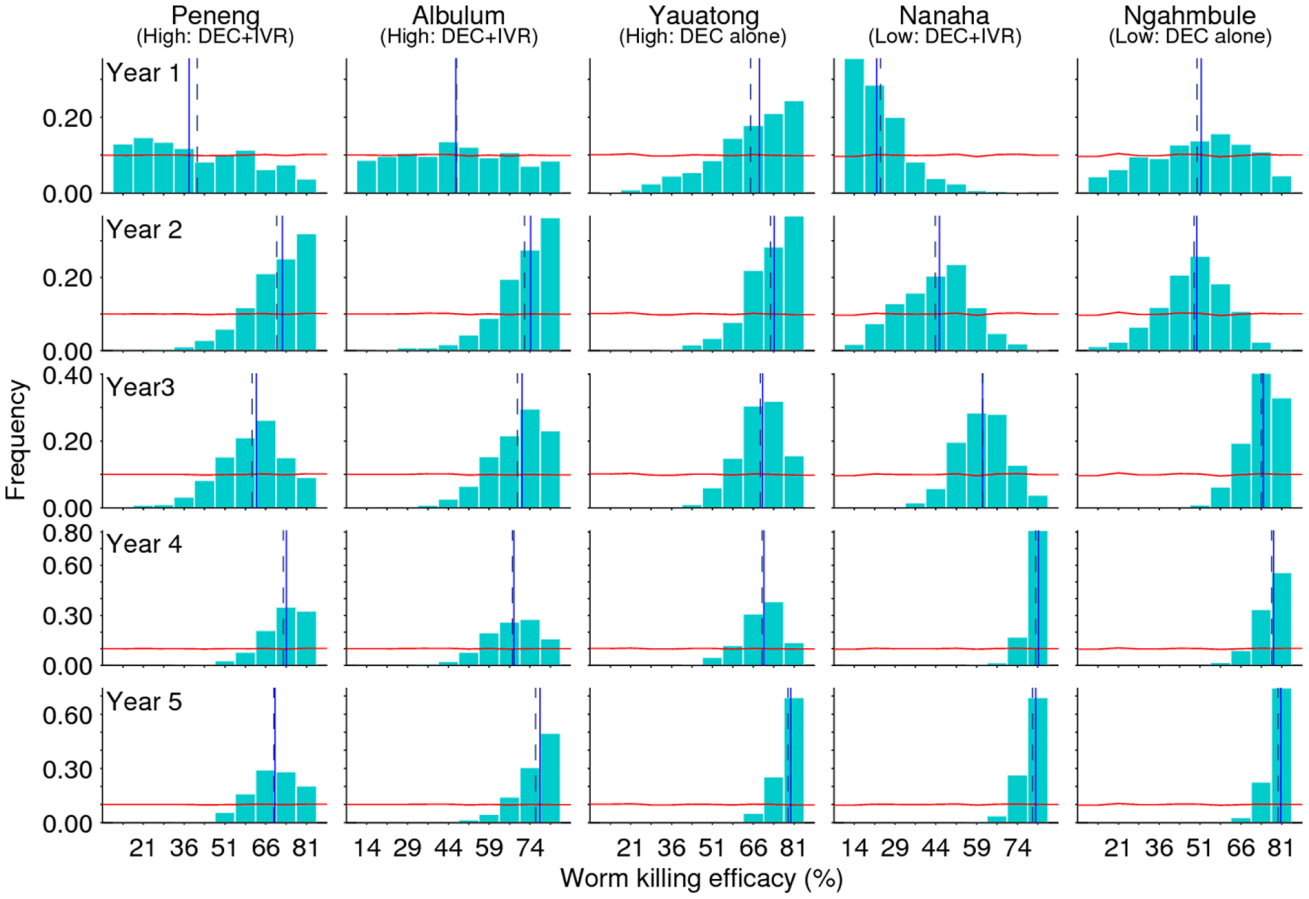
Peak shift in the estimated worm killing efficacy rate of the two drug regimens. Horizontal lines depict the prior parameter distribution, which was assigned to vary from 10% to 85%. Bars represent the estimated relative frequencies of the parameter obtained by annual sequential model fits to data through the 5-year intervention period in each of the 5 PNG study villages. The vertical lines represent measures of the central tendency of the estimated posterior distributions: mean (broken line) and median (solid line).

As noted above, the LF drug efficacy related parameters are (1) instantaneous worm killing efficacy**:** this is major parameter with values assigned previously based on either on expert advice [7], or limitedly estimated from the field data using a mechanistic transmission model (see [26]); (2) microfilariae killing efficacy: better information available from published community trials where values from 69.8% to 91.1% have been demonstrated previously [30], [60]; (3) suppressed production of mf by worms surviving MDA, and (4) the waning period: this is the short time-period that follows the commencement of mass drug administration during which the drug is assumed to remain effective, which is expected to range from between 3 to 9 months [7]. The summary statistics of their estimated posterior parameter distributions in this study indicate gains in knowledge for these parameters from the sequential updating of the LF model, with all parameters in general showing shifts from their assigned prior distributions as interventions progressed (**Figure 5**, **Figures S2, S3, S4** and **Table S3** in Supporting Information S1). The biggest and most informative posterior distribution shift, however, occurred for the worm killing efficacy parameter, with efficacy rates increasing to apparently settle around a peak mean rate of 74 to 77% (**Figure 5**). Mean efficacy rates also increased with sequential model updates for the microfilariae killing and waning period parameters to approximately 80% and 4 months respectively by year 5 post-intervention, although these increases were not found to be statistically significant. The least change occurred for the worm fecundity reduction parameter. Interestingly, the addition of IVR to DEC did not appear to significantly change the model estimated drug efficacy parameter distributions indicating that the major impact on reducing LF infection using the combination therapy is due to the effects of DEC. By contrast, an intriguing finding was that the mean worm killing rate became marginally higher with increasing cycles of intervention in the villages exposed to the low (Nanaha, Ngahmbule) compared to the high (Peneng, Albulum, Yauatong) transmission intensity (**Figure 5**).

### Backfitting to Reconstruct LF Baseline Data

**Figure 6:**
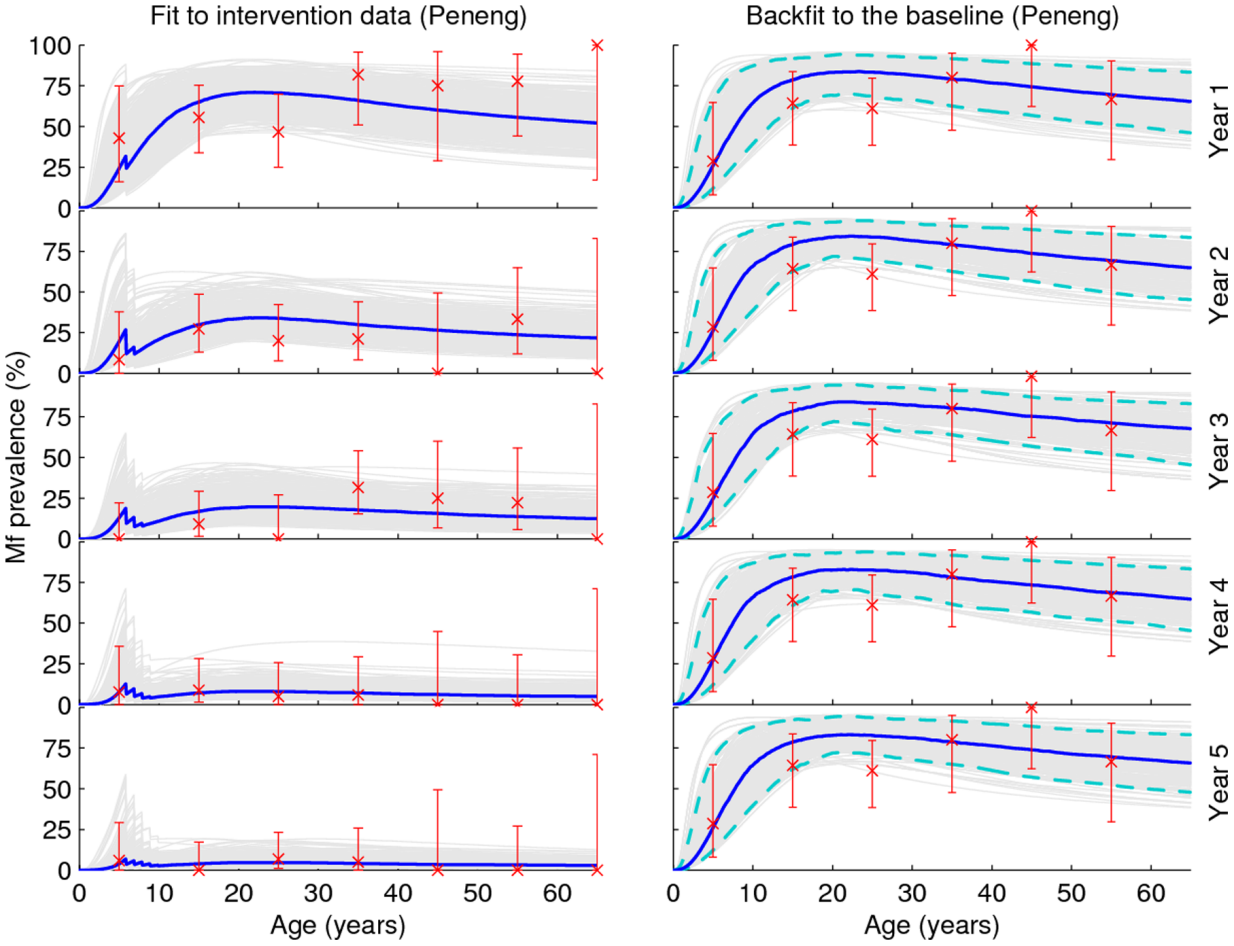
Sequential model backfitting to baseline infection data. Comparisons of sequential model fits to observed annual declines in mf age-prevalence (left panel), and of back predictions of each yearly fit to observed baseline infection data for the village of Peneng (right panel). Sequential parameter updating was accomplished by using posteriors from the model fit to a previous year as priors for each successive subsequent year, starting with the posteriors obtained by model fitting to year 1 intervention data (see text). The thick (blue) line represents the median value of the SIR selected 500 prevalence curves. The observed annual declines in mf age-prevalence (left panel) and baseline mf age-prevalence (right panel) respectively are shown by crosses with 95% CIs. The dashed lines in the right-panel plots represent the 95% bounds (the 2.5^th^ and 97.5^th^ percentile values) of the simulated mf-prevalence curves shown in grey. Similar results were obtained for the remaining 4 study villages (**Figures** **S5, S6, S7,** **S8** in Supporting Information).

We carried out this analysis to test the hypothesis that if the biological parameters of the model (*i.e.* not drug efficacy parameters) remained stable or are invariant during the intervention period for a site, then it will be possible to use the sequential fits to reconstruct that site’s baseline age-infection profile. The mf-prevalence curves generated by sequential model fitting/updates to the age-infection data following each of the five annual drug interventions from year 1 post-intervention (left panel), and their usefulness for reconstructing baseline infection (right panel) are shown in **Figure 6** for the village of Peneng (similar findings were obtained for all the rest of the study villages and are shown in **Figures S5, S6, S7, S8** in Supporting Information S1). All of the individual sets of *l* ( = 500) parameter vectors were used to simulate the baseline age-profiles of mf-prevalence, and the posterior individual curves, medians, and their 95% credible intervals from these simulations are plotted in the figure. The results depict that, although there are year-to-year variations in the backfitted age-mf profiles, the sequentially fitted parameter vectors from each individual year are able to recover the baseline situation remarkably well. **Table 3** provides the age-stratified and overall Monte Carlo *p-*values for the fits of each the backward extrapolations to the example Peneng baseline infection data. The results indicate that model performance for recovering the baseline LF infection for this study site was excellent, when evaluated using age-aggregated data whereas when assessed against age-stratified baseline data discrepant results were obtained for only the youngest 0–10 age group. Similar results were obtained for the rest of the study villages (**Table S4** in Supporting Information S1).

**Table 3:** Carlo *p*-values for the backfitted models to the Peneng baseline data.

	Age-stratified and overall Monte Carlo *p*-values						
Year	0–10	10–20	20–30	30–40	40–50	50–60	Overall
*	0.735	0.841	0.998	0.112	0.998	0.213	0.988
1	0.055	0.842	0.996	0.118	0.996	0.199	0.984
2	0.034	0.84	0.998	0.13	0.998	0.166	0.978
3	0.082	0.812	0.998	0.056	0.986	0.277	0.98
4	0.018	0.854	0.999	0.186	0.999	0.164	0.98
5	0.046	0.841	0.998	0.112	0.998	0.213	0.988

(*) The *p*-values are calculated for the model fits directly fitted to the baseline data. The numbers in the first column represent the intervention years. In this case, the posteriors of the non-drug parameters, obtained from the sequential model fits to those years’ post-intervention infection data, were used to reconstruct the baseline.

### Worm Breakpoint Comparisons

**Figure 7:**
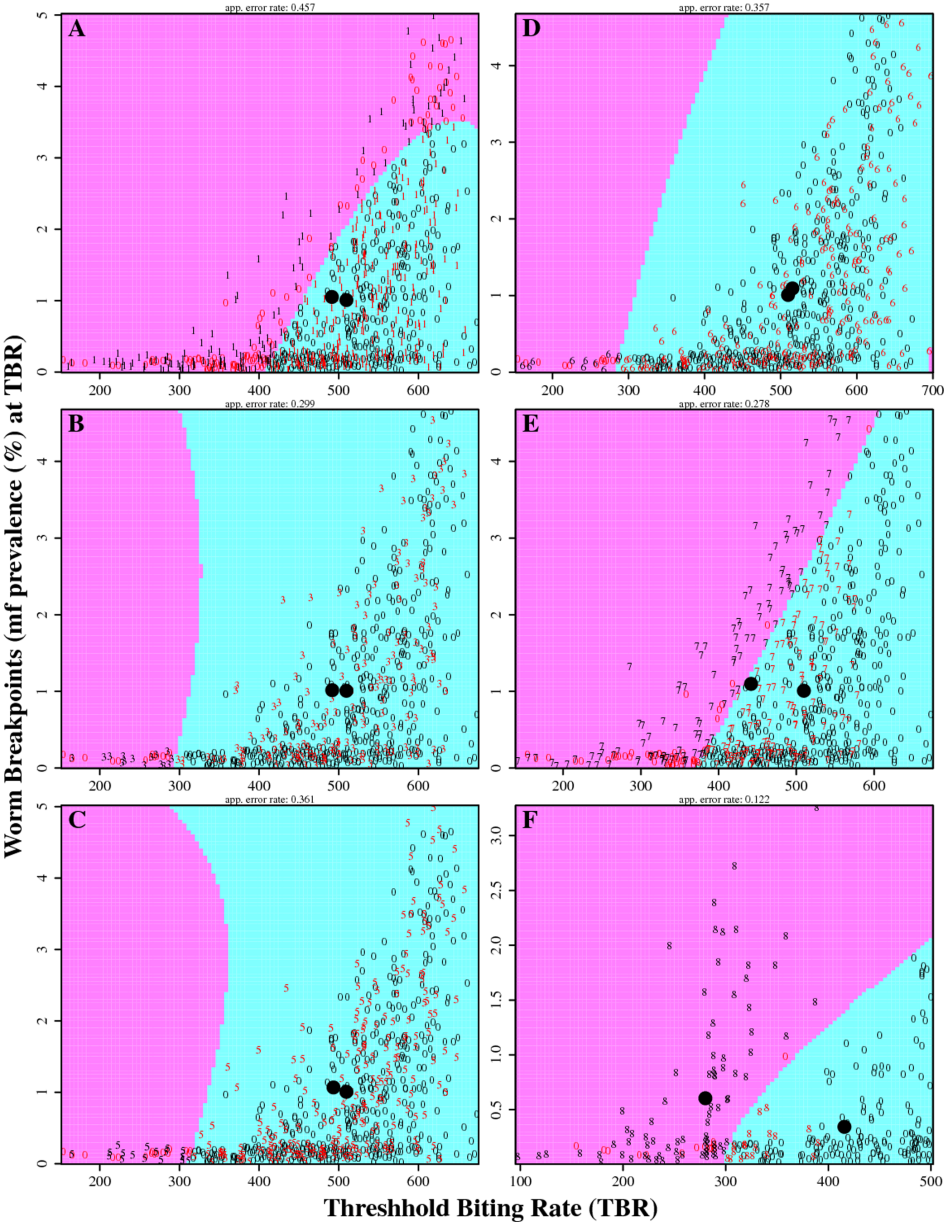
Comparison of results from carrying out a quadratic discriminant analysis on worm breakpoints and threshold biting rates estimated from direct- and back-fitted LF models to baseline mf age-prevalence data for Peneng village. In (A) the endpoint data (worm breakpoints and biting thresholds) estimated by direct model fits to the baseline mf age-prevalence data (shown by **0**) are compared against endpoint estimates derived from the reconstructed baseline mf age-prevalence obtained via model fitting to the first year intervention data (shown by **1**). Similarly, in (B) and (C) the directly fitted model estimates are compared against those obtained from backfitted mf age-prevalences using, respectively, the third (denoted by **3**) and fifth (denoted by **5**) years’ intervention data. In the right panel, the endpoint estimates from the model fitted directly to baseline infection data are compared against those estimated from the backfitted models using the fifth year intervention data, but with the original annual biting rates increased by 25% (shown by **6**) in (D), reduced by 25% (shown by **7**) in (E), and by 50% (shown by **8**) in (F). The edges between the two coloured regions in each graph depict the separation boundaries for the direct and back-fitted endpoint estimates in each case. The two solid dots in each plot depict the means of the distributions of the endpoint estimates obtained from the direct- and the back-fitted models to the baseline data. Note that the two dots begin separating from each other but still remaining in the same coloured region in (E), with full separation between the two estimated distributions (*i.e.* falling in a distinct coloured region) occurring only in (F).

**Figure 7** depicts and compares the mf breakpoint values obtained from the stability analyses of the directly (*i.e.* fitted only to baseline data) versus 1) sequentially fitted LF models to the year 1, 3 and 5 post-MDA data, and 2) a situation reflecting either a 25% or a 50% overall reduction in initial annual biting rate over the entire 5-year MDA period, both for the study village of Peneng. The results from the plotted quadratic discriminant analyses show clear separation between breakpoint values estimated retrospectively using backfitted models compared to those calculated from the direct fits to baseline data only in the case when the initial biting rate is reduced by 50% over the entire MDA period. Reduction of annual biting rate by 50% also raised worm breakpoint values and generated lower threshold biting rates (**Figure 7F**). The formal KS test underscored these results indicating a significant difference (*p* = 0.0042) only for the direct versus backfitted estimates in the case of the 50% reduction in the biting rate scenario. Similar findings were also obtained for the rest of the study villages (data not shown).

## Discussion

In our previous work, we had highlighted the importance of assimilating locality-specific LF infection data into process-based mathematical models as a strategic framework for determining LF elimination endpoints that best reflect the realities of transmission in local environments [12]. Here, we extend that work by investigating if the biological parameters underlying LF transmission estimated by calibrating mathematical models using baseline pre-intervention data in a field site are robust to infection perturbations induced by drug regimens applied sequentially at that site. This is an important question of practical significance to the current drug-based LF elimination program, because if these model parameters are found to be dynamically variable then parasite infection dynamics, including endpoint values, are likely to also vary as interventions proceed. This would make predictions of when elimination may be achieved commensurately more difficult, besides indicating a need for adaptively revising intervention schedules (*e.g.* duration, frequency of MDAs) periodically as programs progress. On the other hand, if the parameters in a site undergoing interventions are found to be stable in the face of perturbations, then this robustness would likely enhance achievement of LF elimination since it would imply that endpoints estimated from either baseline or monitoring data during interventions are exchangeable, and therefore either could serve as reliable targets for determining parasite extinction.

The major result of this study viz. that the biological parameters underpinning LF transmission may indeed remain dynamically stable at least over a moderately long MDA program (5 years) in individual endemic localities, may therefore represent an outcome of import to the current LF elimination initiative. This result suggests: 1) that LF extinction dynamics and hence elimination endpoints in different sites are unlikely to vary over the durations of MDA expected to bring about parasite elimination in most endemic communities [7], [12], 2) that endpoint values may be estimated equally reliably from either baseline pre-MDA or from intervention monitoring data depending on data availability in a site, and finally, 3) that monitoring data during a MDA may be used to reliably back calculate or hindcast initial baseline age-infection patterns, thereby allowing generation of the expected system trajectories required for evaluating the progress made towards LF extinction for all those sites without such data [27], [29]. Taken together, these new findings enhance the prospects of LF elimination as they signify that once endpoints and original infection are known/estimated for a site, initially specified optimal intervention schedules and parameters (*e.g.* coverage/duration of MDA) for crossing such endpoint targets may be followed without alteration until the end of the program, thereby significantly simplifying the management of parasite control [27], [29].

The finding of temporal invariance or dynamic stability in key biological parameters in this study has also shed intriguing new insight into the nature of LF transmission in endemic communities. In particular, it supports recent work exploring the design of biological systems, which suggests that such invariance in biological parameters could be a direct function of the structural adaptation of a complex biological system, such as LF, to initial ecological conditions of a locality so that maintenance of system function (parasite transmission in the present case) against perturbations are preserved locally [19]–[23], [61]. This perspective suggests that the remarkable observed persistence of LF transmission in the face of the drug perturbations carried out in the PNG study sites could be a direct outcome of the complex biological architecture which underpins the transmission of vector-borne diseases - viz. diverse regulatory feedback loops that are able to compensate for changes in ecological conditions, diversity and modularity in overall transmission function by involving two hosts and heterogeneous mosquito larval habitats, which can help contain perturbations locally to minimize effects on the whole system, and different parasite stages exhibiting markedly different life spans to buffer interactions against perturbations [21], [23] - adapted to be robust to normal variations in the values of key transmission-related ecological factors prevailing in a given locality. This implies that if LF transmission parameters are stable, then initial conditions will set the boundaries of local transmission dynamics [21], [23], [61]. Recent studies in schistosomiasis modelling have also highlighted the impact and need for estimating site-specific internal determinants of parasite transmission for gaining a better understanding of the population dynamics and control of *Shistosoma japonicum* in endemic communities [52], [56], [62]. This finding, together with the present results, indicate firstly that robustness to initial conditions in a site may be a fundamental structural feature of vector-borne, and possibly other, parasitic transmission systems, and secondly that gaining a better understanding of the mechanisms underlying such dynamics will be fundamental to identifying the faults and hence the effective countermeasures required to disrupt parasite transmission reliably.

However, it is clear that a trade-off arising from such dynamics is that locally robustly adapted systems may also face devastating fragilities in the face of novel environmental conditions or perturbations leading to catastrophic system failure [21], [23], [61]. Here, we have demonstrated that one major initial external factor that may confine LF dynamics over time in an endemic locality is vector abundance (**Figure 7**). The available data on changes in entomological variables over the 5 year MDA period in each village showed yearly fluctuations that were most prominent for the high transmission villages studied here [30]. Our analysis of the impact of these fluctuations depicted in **Figure 7** shows that LF transmission robustness could be sensitive to such vector abundance changes, but only when >50% change occurred in this key variable over an intervention period. By contrast, when fluctuations in vector biting rates came close to but not >50% (as actually recorded for the high transmission study villages examined here [30]), LF transmission robustness was maintained. This finding indicates that model calibration to key local variables, such as vector biting rates, may allow detection of the boundaries or thresholds of the specific initial constraining conditions that confine dynamics to a site. A major question in this regard is then whether once the values of this confining variable traverse such thresholds, this change might shift the transmission dynamics (and associated attractors, basins of attraction and stability) of the historically adapted LF system into a new and possibly more fragile transmission state and push the system into extinction [20]. Our investigation of this question with regard to changes in vector biting rate has indicated that this outcome might be a distinct possibility in the case of LF. This is illustrated in **Figure 7F**, which shows that if the initial bounding vector abundance variable in a site is reduced over time by a large degree (>50%), fragile LF transmission regimes may indeed emerge as demonstrated by the shifts to higher worm endpoints and lower TBR values in the figure. This finding presents a new mechanism in support of our previous conclusion from modelling intervention dynamics regarding the role of vector control in LF elimination, viz. that including vector control into MDA programs can significantly accelerate the achievement of parasite elimination by altering both vector transmission and worm breakpoint thresholds negatively [7], [9], [13].

The sequential BM fitting of the anopheline-mediated LF model to longitudinal mf data from each of the study villages has also shown that the temporal invariance observed for the biological parameters of this model was compensated for by the occurrence of significant changes in all of the corresponding drug-related parameters relevant to modelling the impact of the drug regimens used in each site, viz. DEC alone or combined DEC/IVR. The estimated posterior distributions for each of four drug parameters –1) worm killing efficacy, 2) mf killing efficacy, 3) reduced fecundity of surviving worms and 4) the waning period – showed variable shifts from their initially assigned prior distributions in each site, with the most informative change observed for the worm killing efficacy parameter, the mean rates of which appearing to settle around values of between 74 to 77% for the two drug regimens by the end of 5 MDA cycles in these study villages (**Figure 5**). Similarly, mean efficacy rates for mf killing and the waning period parameter increased to peak values of around 80% and 4 months respectively over the same intervention period. These sequential model estimates indicate that while previous estimates of the mf killing and waning rates for the present drug regimens appear to have been generally well defined [7], [26], the worm killing rate, by contrast, has been grossly underestimated, suggesting that previous modelling results of the impact of MDA may have been overly pessimistic. Furthermore, the results also show that the addition of IVR to DEC does not appear to markedly change the estimate for this parameter in comparison to the effects of the DEC only regimen. Thus, our results indicate that the major impact of the combination therapy in reducing LF infection in field settings is attributable mostly to the effects of DEC. This finding implies that regimens with IVR but without DEC, such as the ivermectin/albendazole (IVR/ALB) combination regimen advocated for use in many parts of Africa with co-occurring onchocerciasis [1], will have a comparatively lower impact - indeed there is some evidence that ALB would also further lower the efficacy of this combination by an inhibitory effect on worm killing [63] - in reducing LF transmission, thus making parasite elimination more difficult to achieve in such areas. Note that such an inhibitory effect may also reduce the worm killing efficacy of DEC in the DEC/ALB combination, which would clearly have implications for LF elimination in other areas; more biological details of this effect or data from sites using the DEC/ALB combination regimen will need to be modeled if we are to better estimate such inhibitory effects. A further complication highlighted by our results regarding the effectiveness of MDA is that there may also be a density-dependent effects on worm killing by both the applied DEC and DEC/IVR regimens, with the mean worm killing rate estimated for each regimen being marginally lower at each cycles of intervention in the villages exposed to the high compared to the lower transmission intensity (**Figure 5**). The import of this result is clear: worm killing efficacy of anti-filarial drug regimens will vary with the intensity of infection in a community. These findings further emphasize the crucial requirement for estimating transmission and intervention parameters that reflect local realities of transmission if LF is to be reliably eliminated from endemic communities. They also underscore the importance of including supplementary measures, such as vector control, to MDA, this time both to overcome the effects of using a less efficacious macrofilaricidal regimen in DEC contra-indicated regions and to overcome the possibility of density-dependent worm killing effects, if we are to achieve LF elimination globally.

Our use of the BM methodology to integrate sequential intervention-related infection data with the LF model to predict the effects of MDA on infection dynamics show that this Monte-Carlo based Bayesian calibration method can yield results that efficiently approximate reality as close as possible. As highlighted previously [51], [54], the innovative benefit using this approach rather than simple Monte Carlo simulations lies in the ability to attach prior distributions and calculate likelihoods for both model input and the output, which improves the use of data to inform initial conditions, update model parameters, and constrain a model during simulation so to match observed data closely. However, one drawback of using sampling filters such as the SIR algorithm used in this study to generate the posterior probability distributions of model parameters and predicted state variables (*e.g.* endpoints) is that it may lead to sample impoverishment, wherein the posterior sample may have a large number of repeated copies due to the weighted resampling carried out based on likelihoods of parameter vectors [64]–[66]. Although this did not represent a major problem in the present study, future applications of this approach to extended datasets and perhaps fitting of models to data from longer MDA cycles than studied here will need to take this technical issue into consideration. Parasite system dynamics, particularly in the case of vector-borne diseases, are also likely to be strongly influenced by weather and climate changes via effects on vector population dynamics. As shown in this study, such vector abundance changes can critically impact parasite transmission dynamics in a locality, meaning that future studies may require considering statistical frameworks, such as Markov Chain Monte Carlo (MCMC) methods or hybrid sequential Monte Carlo sampling/MCMC approaches, for joint estimation of time varying parasite system states and parameters [57], [64], [66], [67]. We are currently extending our LF model to explicitly include mosquito population dynamics (rather than using a fixed ABR value to represent such dynamics as performed here) as a first step to developing such statistical methods. Note also that if it is confirmed that local LF transmission dynamics can be largely constrained by the prevailing vector abundance in a site, another outcome of such a finding is that it may also allow the application of our model, and hence transferability of results, across different settings based on calibration to available joint spatial data on vector biting rates and mf prevalences.

A further improvement to our modelling approach is to directly include infection diagnostic uncertainties into the model calibration approach so that true prevalences can be quantified from joint estimates of test specificities and sensitivities [68], [69]. This will clearly be important in correcting for endpoint values estimated using different types of infection indicators as well as for correctly predicting the corresponding system trajectories due to the application of MDA in different localities [27]. Such work will improve understanding of how indicator dynamics are linked to the underlying parasite transmission dynamics, and in turn guide the choice of the best indicators that closely match worm dynamics for monitoring the impact of interventions on LF infection/transmission in communities [27], [29].

Despite these limitations related to our current model and its calibration, the present study has nonetheless advanced new understanding regarding LF transmission and the prospects for its control using currently available MDA methods. Firstly, our findings underscore the need to understand the biological robustness of parasite transmission, and the factors that underlie such robustness, in local settings if we are to develop reliable countermeasures to achieve parasite extinction. Our preliminary analysis of LF transmission robustness to initial conditions has highlighted the impact that fluctuations in vector abundance can play in pushing a locally robustly adapted vector-borne parasite system to a different state with its own dynamics, and hence endpoints. This result corroborates previous theoretical and experimental work in demonstrating that adaptability or robustness of biological systems, including complex parasitic systems, to one set of conditions can enhance their fragility to abrupt changes in such conditions [19]–[23], [61]. We suggest that learning about these trade-offs between transmission robustness and fragility has now become central if we are to successfully accomplish the elimination of LF, and indeed other parasitic infections, both locally and globally. Secondly, results presented here also suggest that progress in this next generation of work will not only require the development of modelling methods to analyse and evaluate biocomplexity and robustness of parasitic systems, but also the development of data-driven inferential techniques that can reliably allow the joint determination of model states, structure and calibration using local data. A key need here is the development of modelling frameworks that can assimilate spatially distributed data on key ecological conditions relevant to parasite transmission from a variety of sources, couple with climate and weather dynamics, and integrate with the available spatio-temporal datasets on human infection prior, during and following interventions [57], [67]. Constructing such models of everywhere is no doubt a daunting challenge, but we suggest that with the rapid improvement in computing power, advent of new intelligent eco measurement devices and observation networks, and development of new flexible state space data modelling and assimilation approaches, these challenges can be met [57], [67], and the notion of using models as an effective management tool for achieving parasite control or eradication becomes an attractive, acceptable option. Lastly, a theme that can be identified running throughout this work is a need for control programs to focus on obtaining good quality infection monitoring data. Data will be required to characterize local dynamics, to evaluate and update model predictions, extrapolate predictions to sites without or poor quality data, and monitor changes in coverage and system response as interventions progress. We end by indicating that a closer alignment of modelling work with well-collected field data constitutes an urgent operational requirement if the current global program to eradicate LF is to successfully achieve its laudable goal.

## Supporting Information

Supporting Information S1 File contains eight figures and four tables: **Figure S1 - Predicted age-profiles of mf-prevalence (curves) from model fits to observed baseline and longitudinal post-intervention infection data for the high transmission village of Albulum (DEC+IVR), and the low transmission villages of Nanaha (DEC+IVR) and Ngahmbule (DEC alone).** The observed data points (*crosses*) with 95% binomial credible intervals are shown at the mid-points of each population age-group. Individual 500 best-fit model simulations are shown in grey while the thick blue line represents the median value of these curves. **Figure S2 - No significant changes in the estimated mf killing efficacy rate over time.** Horizontal lines denote the frequency distribution of the parameter prior, which was assigned to vary from 55% to 95% in each village. Bars represent the relative frequencies of the parameter posteriors obtained from the model fits to the infection over the intervention period. The vertical lines depict measures of the central tendency of the estimated posterior distributions: mean (broken line) and median. **Figure S3 - No significant changes in the estimated worm fecundity reduction rate.** Horizontal lines and bars are as described in the previous figure. The prior distribution was set to vary from 55% to 95% in each village. The vertical lines are the estimated means and medians of the posteriors. **Figure S4 - No significant changes in the waning period.** Horizontal lines and bars are as described before. The prior distribution of the waning period was set to vary from 1 to 6 months in each village. The vertical lines are the estimated means and medians of the posteriors. **Figure S5 - Sequential backfitting to baseline data of the village Albulum.** As shown in Figure 6 in the main text, the thick (blue) line represents the median value of the SIR selected 500 prevalence curves. The observed annual declines in mf age-prevalence (left panel) and baseline mf age-prevalence (right panel) respectively are shown by crosses with 95% CIs. The dashed lines in the right-panel plots represent the 95% bounds (the 2.5^th^ and 97.5^th^ percentile values) of the simulated mf prevalence curves shown in grey. **Figure S6 - Sequential backfitting to baseline data of the village Yauatong.** Descriptions of the data shown (denoted by symbols, bars and curves) as given in the legend to Figure S5. **Figure S7 - Sequential backfitting to baseline data of the village Nanaha.** Descriptions of the data shown (denoted by symbols, bars and curves) as given in the legend to Figure S5. **Figure S8 - Sequential backfitting to baseline data of the village Ngahmbule.** Descriptions of the data shown (denoted by symbols, bars and curves) as given in the legend to Figure S5. **Table S1 - Monte Carlo** ***p*** **-values for the directly fitted models to the baseline and five post-intervention infection data collected during mass treatment programme for the five PNG study sites. Table S2 - Results of the univariate Kolmogorov-Smirnov (KS) test of differences between the parameter posterior distributions estimated from sequential model fits to baseline and infection data from five successive years.** The *p*-value shows the significance level of the KS test. A *p*-value <0.05 indicates that posterior distribution is significantly different from its prior. Here the parameter posteriors from the models fitted to the baseline were tested against the non-informative priors. The parameter posteriors from the models fitted to the infection data of the first intervention year were tested against those from the model fitted to the baseline data of each study site; the parameter posteriors of the second intervention year against those of the first year; and so on. **Table S3 - Results of the univariate Kolmogorov-Smirnov (KS) test of differences between prior and posterior distributions of drug related parameters.** The *p*-value shows the significance level of the KS test. A *p*-value <0.05 indicates that posterior distribution is significantly different from its prior, which is always the case for the worm killing efficacy parameter.
(DOCX)
