# Correction: Sequential Modelling of the Effects of Mass Drug Treatments on Anopheline-Mediated Lymphatic Filariasis Infection in Papua New Guinea

**DOI:** 10.1371/annotation/54fdf4a6-e2ad-4a3d-bbe8-b28b7a3c5b1e

**Published:** 2014-01-03

**Authors:** Brajendra K. Singh, Moses J. Bockarie, Manoj Gambhir, Peter M. Siba, Daniel J. Tisch, James Kazura, Edwin Michael

In the second equation of the Methods section, the curly theta (between alpha and the left paranthesis) should be replaced by phi. Note this phi should be the same as the one in the expression below Table 2.

The curly phi used in the function for "Adult worm mating probability" in Table 2 should be replaced by the symbol phi in the expression below Table 2.

In the first paragraph of the section "Sequential BM Model Fitting to LF Infection Data Following Drug Interventions", the capital letter pi should be replaced by the letter theta.

The curly theta in the last equation of the "Modelling the Effects of Annual Mass Drug Administration" should be replaced by the symbol phi. Note this phi should be the same as the one in the expression below Table 2. 

