# Supplementary material for: Sequential Modelling of the Effects of Mass Drug Treatments on Anopheline-Mediated Lymphatic Filariasis Infection in Papua New Guinea
Source: PLoS One. 2013 Jun 24;8(6):e67004. doi: 10.1371/journal.pone.0067004 (PMC3691263; doi:10.1371/journal.pone.0067004)
Supplement: Supporting Information S1 — File contains eight figures and four tables: Figure S1 - Predicted age-profiles of mf-prevalence (curves) from model fits to observed baseline and longitudinal post-intervention infection data for the high transmission village of Albulum (DEC+IVR), and the low transmission villages of Nanaha (DEC+IVR) and Ngahmbule (DEC alone). The observed data points (crosses) with 95% binomial credible intervals are shown at the mid-points of each population age-group. Individual 500 best-fit model simulations are shown in grey while the thick blue line represents the median value of these curves. Figure S2 - No significant changes in the estimated mf killing efficacy rate over time. Horizontal lines denote the frequency distribution of the parameter prior, which was assigned to vary from 55% to 95% in each village. Bars represent the relative frequencies of the parameter posteriors obtained from the model fits to the infection over the intervention period. The vertical lines depict measures of the central tendency of the estimated posterior distributions: mean (broken line) and median. Figure S3 - No significant changes in the estimated worm fecundity reduction rate. Horizontal lines and bars are as described in the previous figure. The prior distribution was set to vary from 55% to 95% in each village. The vertical lines are the estimated means and medians of the posteriors. Figure S4 - No significant changes in the waning period. Horizontal lines and bars are as described before. The prior distribution of the waning period was set to vary from 1 to 6 months in each village. The vertical lines are the estimated means and medians of the posteriors. Figure S5 - Sequential backfitting to baseline data of the village Albulum. As shown in Figure 6 in the main text, the thick (blue) line represents the median value of the SIR selected 500 prevalence curves. The observed annual declines in mf age-prevalence (left panel) and baseline mf age-prevalence (right panel) respectively are sho [file pone.0067004.s001.docx]

# Supporting Information S1

##
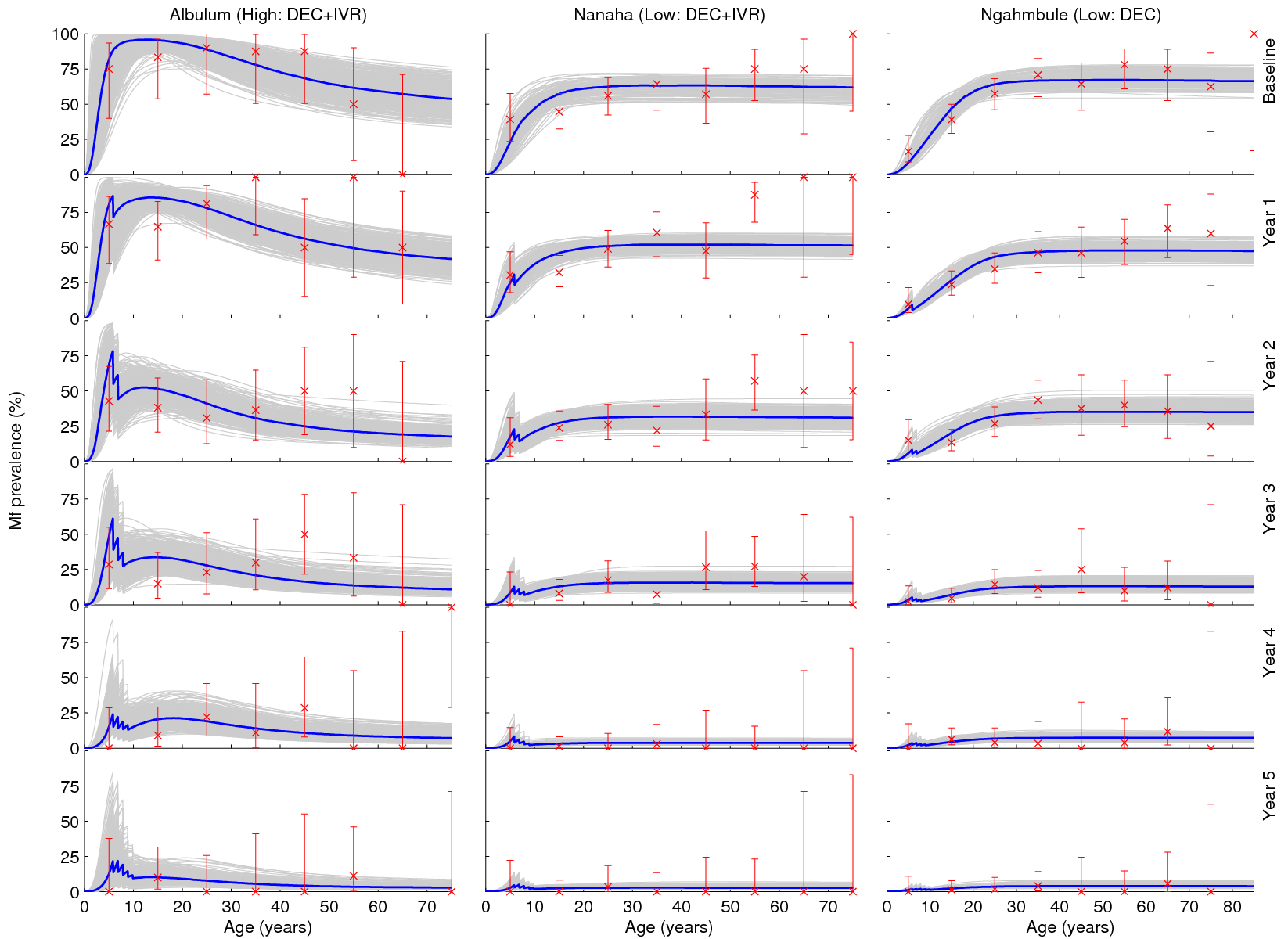


**Figure S1 -** **Predicted age-profiles of mf-prevalence (curves) from model fits to observed baseline and longitudinal post-intervention infection data for the high transmission village of Albulum (DEC+IVR), and the low transmission villages of Nanaha (DEC+IVR) and Ngahmbule (DEC alone).** The observed data points (*crosses*) with 95% binomial credible intervals are shown at the mid-points of each population age-group. Individual 500 best-fit model simulations are shown in grey while the thick blue line represents the median value of these curves.

**
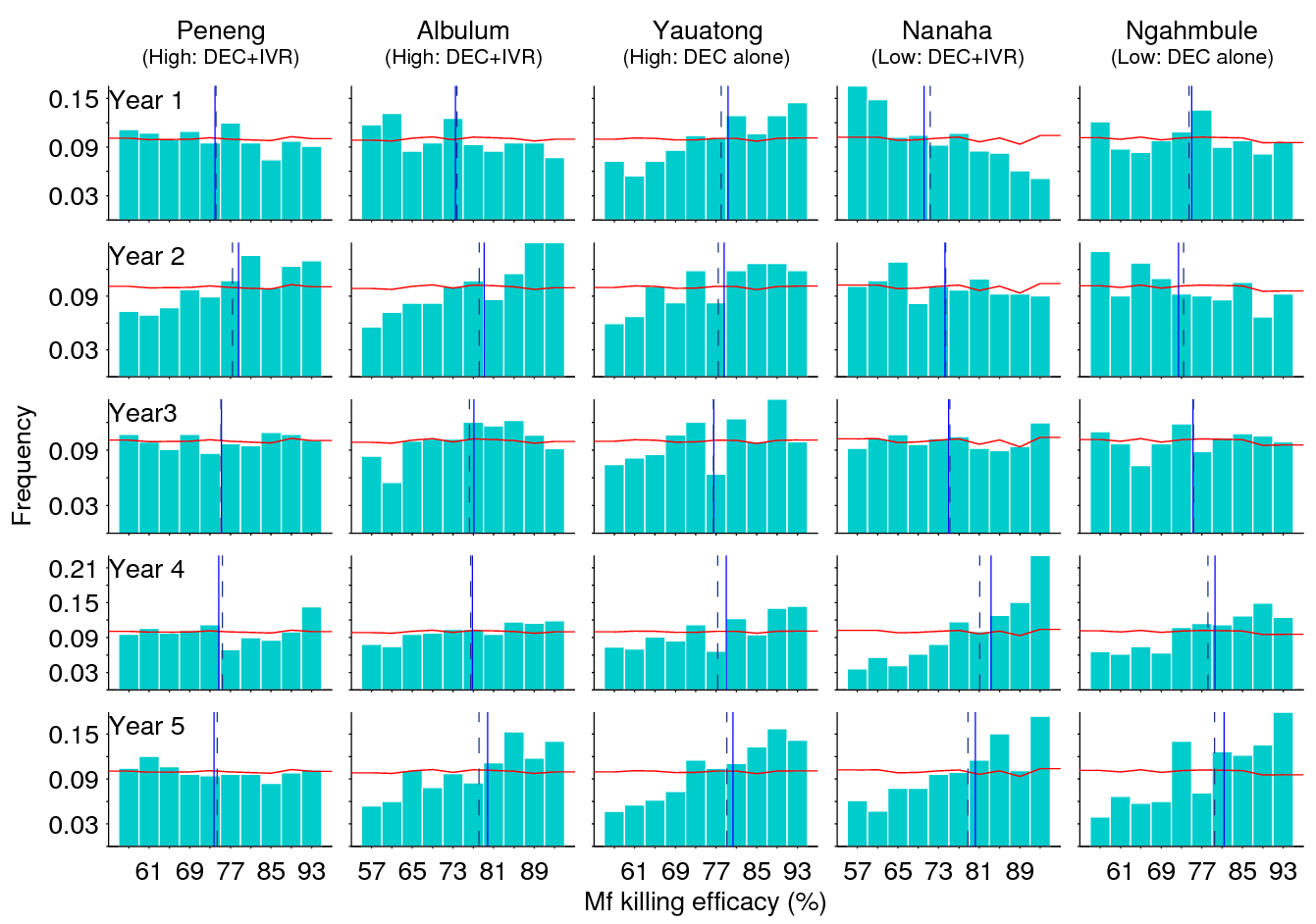
**

**Figure S2 -** **No significant changes in the estimated mf killing efficacy rate over time.** Horizontal lines denote the frequency distribution of the parameter prior, which was assigned to vary from 55% to 95% in each village. Bars represent the relative frequencies of the parameter posteriors obtained from the model fits to the infection over the intervention period. The vertical lines depict measures of the central tendency of the estimated posterior distributions: mean (broken line) and median.


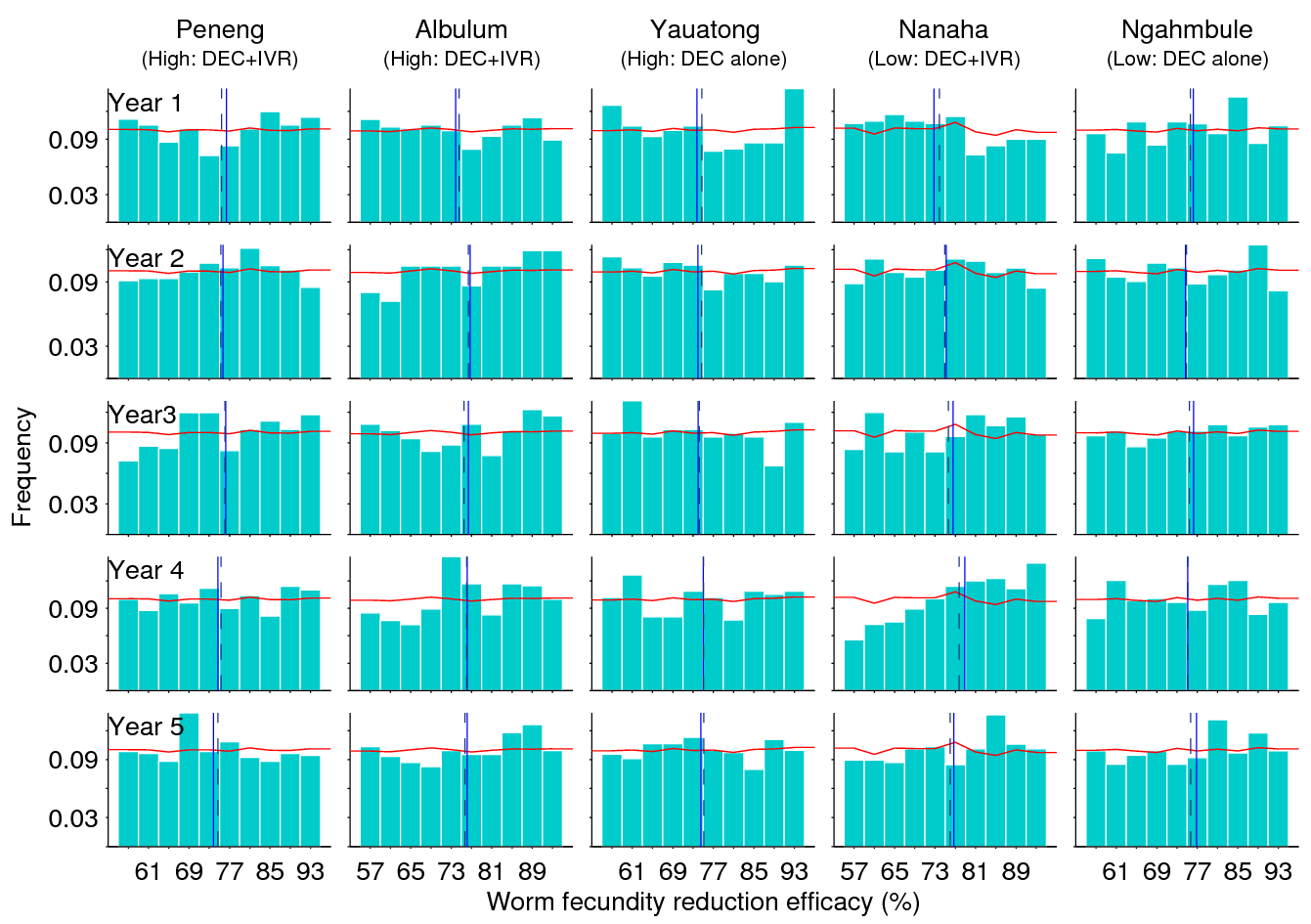


**Figure S3 -** **No significant changes in the estimated worm fecundity reduction rate.** Horizontal lines and bars are as described in the previous figure. The prior distribution was set to vary from 55% to 95% in each village. The vertical lines are the estimated means and medians of the posteriors.


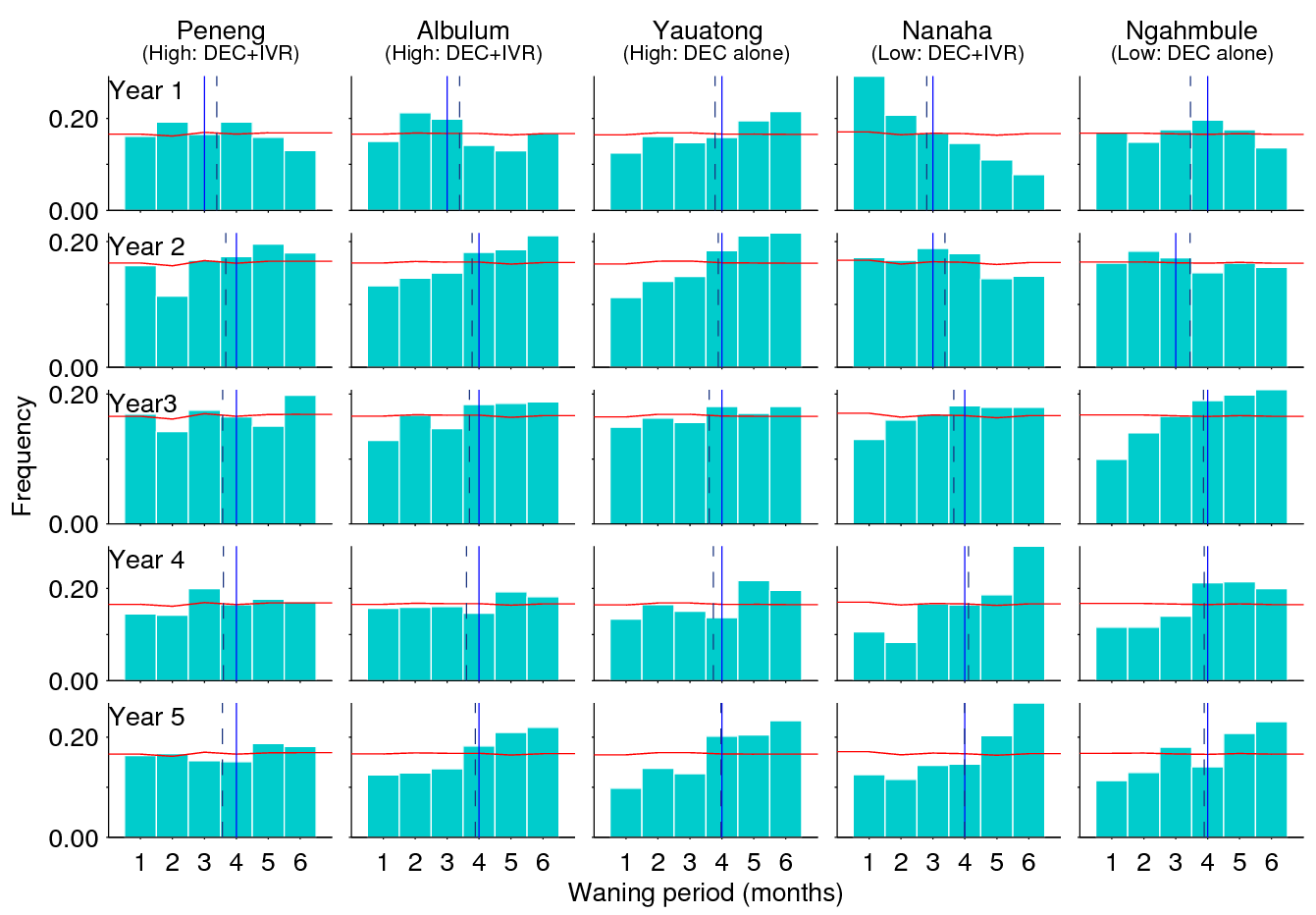


**Figure S4 -** **No significant changes in the waning period.** Horizontal lines and bars are as described before. The prior distribution of the waning period was set to vary from 1 to 6 months in each village. The vertical lines are the estimated means and medians of the posteriors.

**
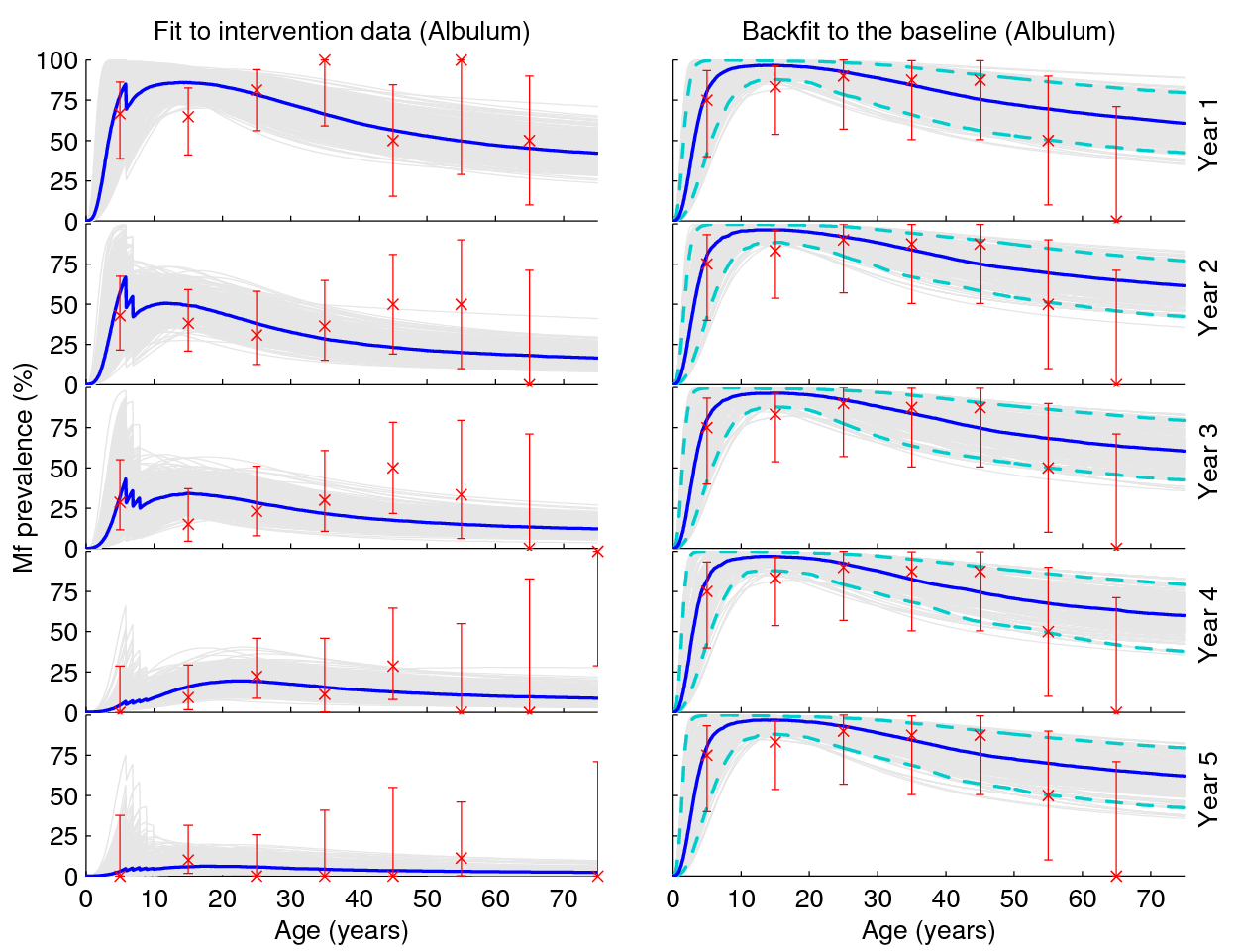
**

**Figure S5** - **Sequential backfitting to baseline data of the village Albulum.** As shown in Figure 6 in the main text, the thick (blue) line represents the median value of the SIR selected 500 prevalence curves. The observed annual declines in mf age-prevalence (left panel) and baseline mf age-prevalence (right panel) respectively are shown by crosses with 95% CIs. The dashed lines in the right-panel plots represent the 95% bounds (the 2.5^th^ and 97.5^th^ percentile values) of the simulated mf prevalence curves shown in grey.

**
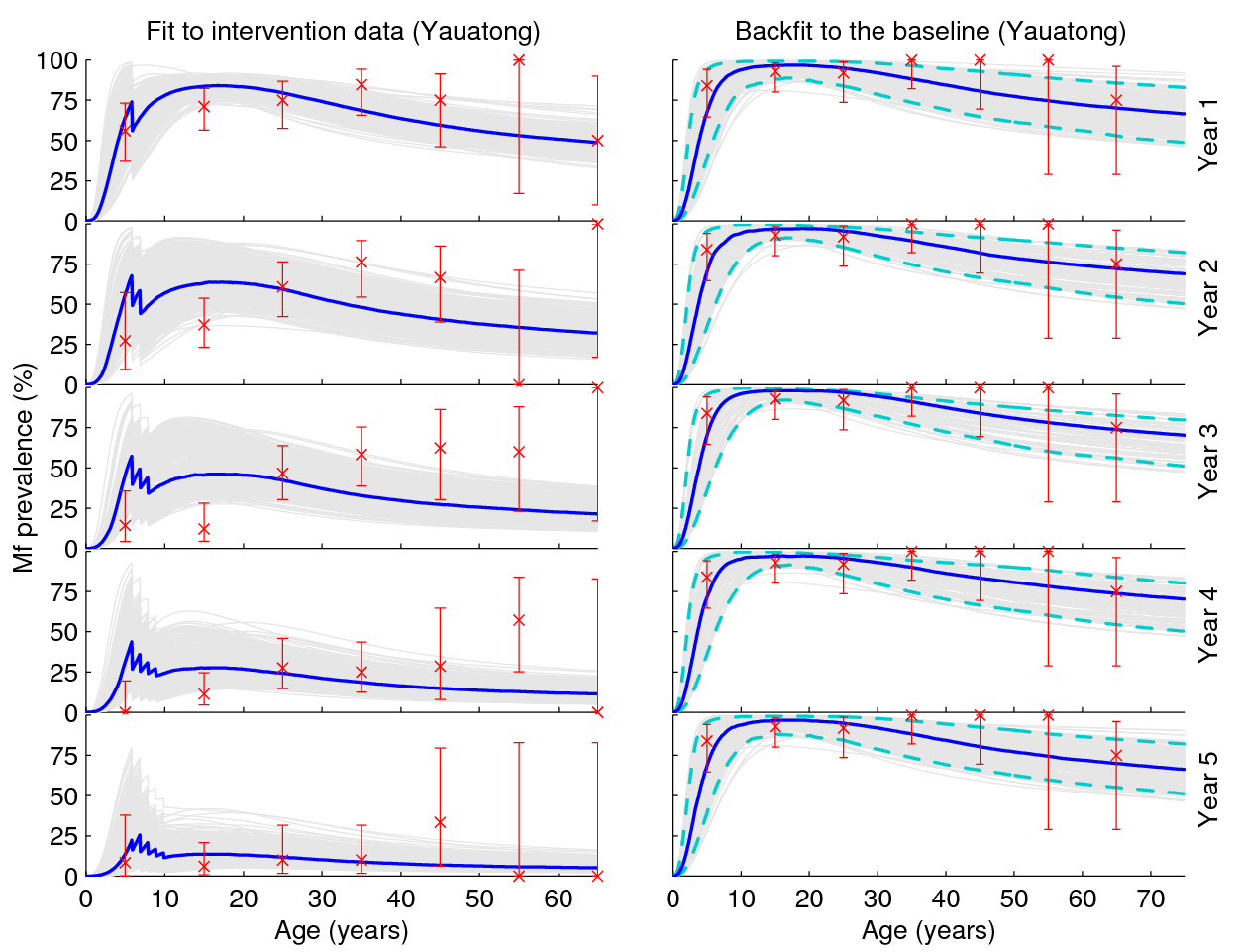
**

**Figure S6 - Sequential backfitting to baseline data of the village Yauatong.** Descriptions of the data shown (denoted by symbols, bars and curves) as given in the legend to Figure S5.

**
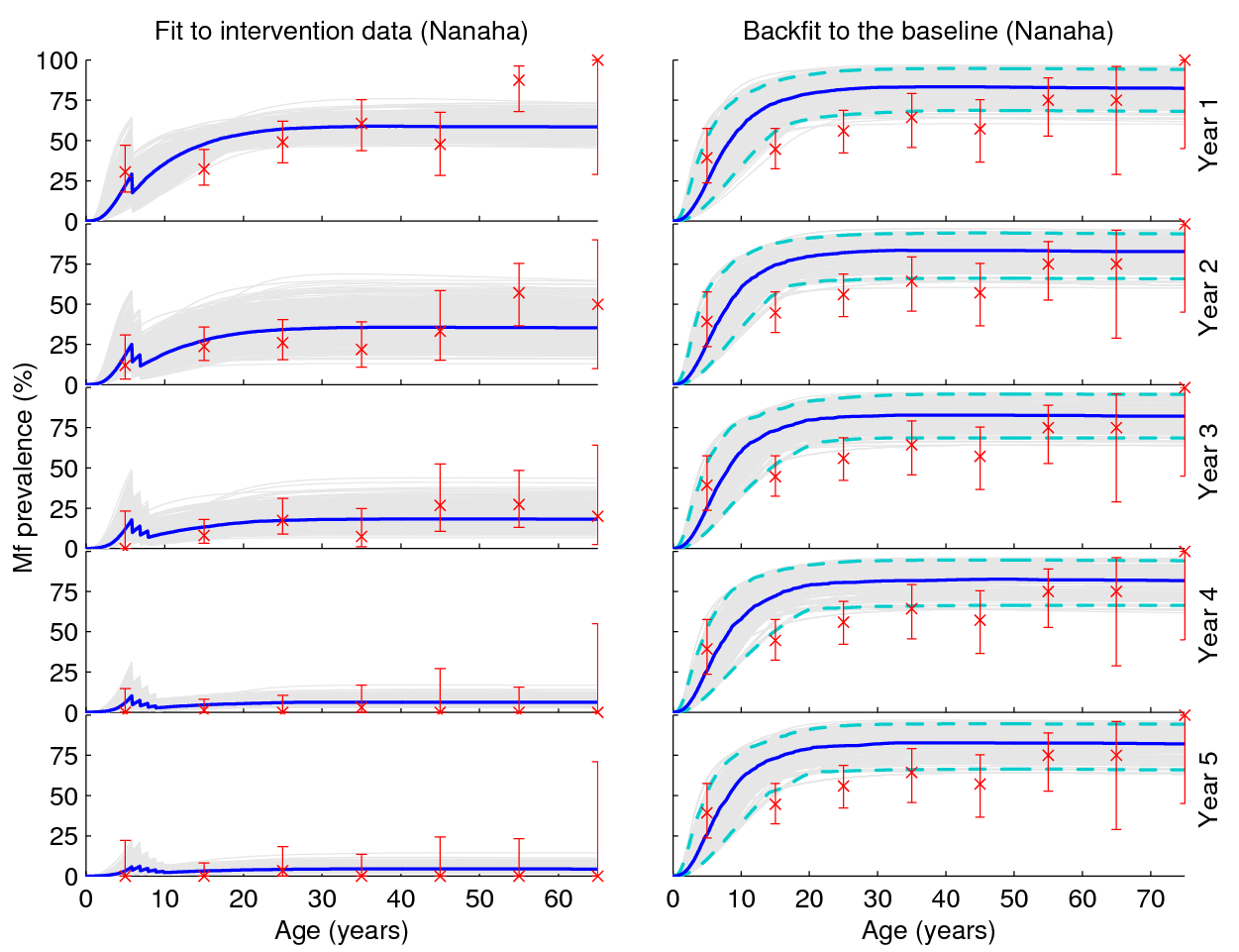
**

**Figure S7 - Sequential backfitting to baseline data of the village Nanaha.** Descriptions of the data shown (denoted by symbols, bars and curves) as given in the legend to Figure S5.

**
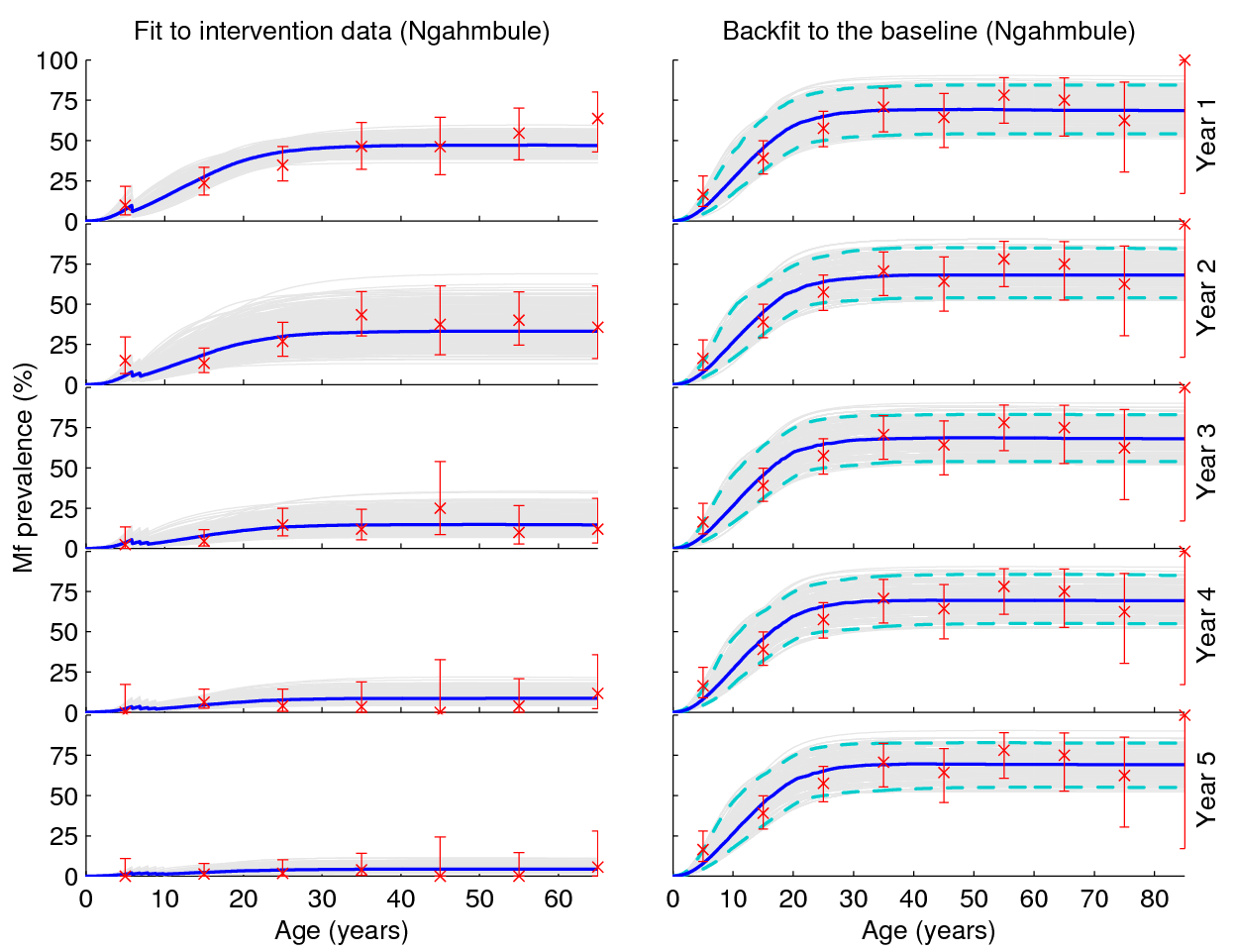
**

**Figure S8 - Sequential backfitting to baseline data of the village Ngahmbule.** Descriptions of the data shown (denoted by symbols, bars and curves) as given in the legend to Figure S5.

**Table S1 - Monte Carlo *p*-values for the directly fitted models to the baseline and five post-intervention infection data collected during mass treatment programme for the five PNG study sites.**

| Age groups:--> | 0 - 10 | 10 - 20 | 20 - 30 | 30 - 40 | 40 - 50 | 50 - 60 | 60 - 70 | 70 – 80 | Overall |
| --- | --- | --- | --- | --- | --- | --- | --- | --- | --- |
|  |  |  | Monte Carlo *p*-values for Peneng | | | |  |  |  |
| Baseline | 0.735 | 0.841 | 0.998 | 0.112 | 0.998 | 0.213 | N/A | N/A | 0.988 |
| Intervention year 1 | 0.999 | 0.152 | 0.998 | 0.994 | 0.971 | 0.996 | 0.999 | N/A | 0.999 |
| Intervention year 2 | 0.018 | 0.518 | 0.921 | 0.67 | 0.999 | 0.996 | 0.999 | N/A | 0.998 |
| Intervention year 3 | 0.978 | 0.737 | 0.998 | 0.992 | 0.937 | 0.909 | 0.996 | N/A | 0.992 |
| Intervention year 4 | 0.918 | 0.699 | 0.695 | 0.184 | 0.973 | 0.975 | 0.975 | N/A | 0.949 |
| Intervention year 5 | 0.974 | 0.96 | 0.716 | 0.3 | 0.956 | 0.954 | 0.954 | N/A | 0.958 |
|  |  |  | Monte-Carlo *p*-values for Albulum | | | |  |  |  |
| Baseline | 0.751 | 0.985 | 0.247 | 0.814 | 0.992 | 0.873 | 0.999 | N/A | 0.998 |
| Intervention year 1 | 0.862 | 0.999 | 0.544 | 0.996 | 0.611 | 0.912 | 0.621 | N/A | 0.994 |
| Intervention year 2 | 0.459 | 0.962 | 0.923 | 0.57 | 0.99 | 0.996 | 0.999 | N/A | 0.999 |
| Intervention year 3 | 0.278 | 0.994 | 0.629 | 0.92 | 0.998 | 0.998 | 0.99 | N/A | 0.99 |
| Intervention year 4 | 0.972 | 0.965 | 0.476 | 0.589 | 0.99 | 0.998 | 0.996 | 0.99 | 0.998 |
| Intervention year 5 | 0.95 | 0.062 | 0.981 | 0.981 | 0.981 | 0.99 | N/A | 0.977 | 0.99 |
|  |  |  | Monte-Carlo *p*-values for Yauatong | | | |  |  |  |
| Baseline | 0.793 | 0.999 | 0.878 | 0.99 | 0.997 | 0.999 | 0.054 | N/A | 0.997 |
| Intervention year 1 | 0.146 | 0.999 | 0.831 | 0.999 | 0.995 | 0.999 | 0.034 | 0.999 | 0.998 |
| Intervention year 2 | 0.871 | 0.999 | 0.218 | 0.999 | 0.999 | 0.997 | 0.999 | 0.999 | 0.999 |
| Intervention year 3 | 0.946 | 0.999 | 0.427 | 0.999 | 0.999 | 0.998 | 0.999 | 0.998 | 0.999 |
| Intervention year 4 | 0.99 | 0.999 | 0.5 | 0.939 | 0.999 | 0.999 | 0.999 | 0.999 | 0.999 |
| Intervention year 5 | 0.73 | 0.982 | 0.484 | 0.505 | 0.998 | 0.998 | 0.998 | 0.998 | 0.999 |
|  |  |  | Monte-Carlo *p*-values for Nanaha | | | |  |  |  |
| Baseline | 0.993 | 0.964 | 0.754 | 0.312 | 0.681 | 0.996 | 0.999 | 0.999 | 0.99 |
| Intervention year 1 | 0.99 | 0.99 | 0.446 | 0.993 | 0.844 | 0.998 | 0.999 | 0.998 | 0.99 |
| Intervention year 2 | 0.232 | 0.591 | 0.836 | 0.994 | 0.396 | 0.999 | 0.999 | 0.999 | 0.999 |
| Intervention year 3 | 0.989 | 0.921 | 0.541 | 0.994 | 0.999 | 0.998 | 0.874 | 0.999 | 0.998 |
| Intervention year 4 | 0.963 | 0.98 | 0.999 | 0.626 | 0.999 | 0.981 | 0.999 | 0.997 | 0.999 |
| Intervention year 5 | 0.979 | 0.991 | 0.514 | 0.988 | 0.988 | 0.988 | 0.988 | 0.988 | 0.988 |
|  |  |  | Monte-Carlo *p*-values for Ngahmbule | | | |  |  |  |
| Baseline | 0.957 | 0.73 | 0.823 | 0.553 | 0.504 | 0.986 | 0.872 | 0.674 | 0.998 |
| Intervention year 1 | 0.996 | 0.608 | 0.994 | 0.023 | 0.253 | 0.96 | 0.998 | 0.998 | 0.998 |
| Intervention year 2 | 0.998 | 0.956 | 0.828 | 0.979 | 0.423 | 0.789 | 0.117 | 0.983 | 0.998 |
| Intervention year 3 | 0.211 | 0.908 | 0.858 | 0.462 | 0.999 | 0.902 | 0.545 | 0.998 | 0.998 |
| Intervention year 4 | 0.975 | 0.962 | 0.966 | 0.975 | 0.999 | 0.969 | 0.973 | 0.998 | 0.991 |
| Intervention year 5 | 0.979 | 0.914 | 0.974 | 0.063 | 0.999 | 0.999 | 0.919 | 0.999 | 0.991 |

N/A represents that there were either no observed data or zero mf-positive cases in the age-group of the study village in question during their baseline and/or post-intervention survey.

# Table S2 - Results of the univariate Kolmogorov-Smirnov (KS) test of differences between the parameter posterior distributions estimated from sequential model fits to baseline and infection data from five successive years. The *p*-value shows the significance level of the KS test. A *p*-value <0.05 indicates that posterior distribution is significantly different from its prior. Here the parameter posteriors from the models fitted to the baseline were tested against the non-informative priors. The parameter posteriors from the models fitted to the infection data of the first intervention year were tested against those from the model fitted to the baseline data of each study site; the parameter posteriors of the second intervention year against those of the first year; and so on.

| Study site: the PNG village Peneng | | | | | | | | | | | | |
| --- | --- | --- | --- | --- | --- | --- | --- | --- | --- | --- | --- | --- |
|  | Baseline | | Intervention Year 1 | | Intervention Year 2 | | Intervention Year 3 | | Intervention Year 4 | | Intervention Year 5 | |
| Parameter  symbols | Median | KS test  *p*-value | Median | KS test  *p*-value | Median | KS test  *p*-value | Median | KS test  *p*-value | Median | KS test  *p*-value | Median | KS test  *p*-value |
| *λ* | 9.47346 | **0.0004** | 9.31819 | 0.90215 | 9.470031 | 0.959646 | 9.338441 | 0.964124 | 9.673507 | 0.397256 | 9.444594 | 0.817802 |
| *α* | 0.838335 | **0** | 0.905072 | 0.23443 | 0.855768 | 0.328609 | 0.898924 | 0.5358 | 0.833923 | 0.205823 | 0.822205 | 0.99997 |
| *k_0_* | 0.000245 | 0.7856 | 0.000246 | 0.966247 | 0.000236 | 0.79144 | 0.000247 | 0.670403 | 0.000225 | 0.382307 | 0.000249 | 0.716981 |
| *k_Lin_* | 0.010352 | **0.0114** | 0.011296 | 0.486342 | 0.011059 | 0.98358 | 0.010871 | 0.99641 | 0.010481 | 0.970267 | 0.010481 | 0.986929 |
| *σ* | 5.31395 | **0.0001** | 5.372627 | 0.992707 | 5.27347 | 0.934054 | 5.27052 | 0.999575 | 5.436183 | 0.849123 | 5.27052 | 0.940145 |
| *Ψ_1_* | 0.348648 | **0** | 0.349211 | 0.877605 | 0.350485 | 0.646832 | 0.32976 | 0.535525 | 0.356561 | 0.380003 | 0.361643 | 0.990928 |
| *Ψ_2_s_2_* | 0.001489 | **0** | 0.001255 | 0.135775 | 0.001483 | 0.150407 | 0.001338 | 0.732145 | 0.00161 | 0.058319 | 0.001509 | 0.377753 |
| *μ* | 0.012167 | **0.0032** | 0.012344 | 0.905521 | 0.012572 | 0.346055 | 0.011904 | 0.14685 | 0.012501 | 0.069222 | 0.012029 | 0.516048 |
| *γ* | 0.100892 | 0.0865 | 0.100926 | 0.999947 | 0.101176 | 0.983505 | 0.100892 | 0.819076 | 0.100398 | 0.946629 | 0.10023 | 0.957148 |
| *g* | 0.359096 | **0.0005** | 0.349998 | 0.933805 | 0.366543 | 0.494201 | 0.359096 | 0.953104 | 0.366354 | 0.95505 | 0.349953 | 0.291942 |
| *c* | 0.000833 | **0.0001** | 0.000674 | **0.002575** | 0.000904 | **0.000099** | 0.000744 | **0.025736** | 0.000951 | **0.003737** | 0.000883 | 0.764553 |
| *δ* | 0.0 | 0.999999 | 0.0 | 0.999997 | 0.0 | 0.999997 | 0.0 | 0.999997 | 0.0 | 0.999997 | 0.0 | 0.999997 |
| *H_lin_* | 13.5065 | **0** | 13.06704 | 0.630097 | 12.13843 | 0.436509 | 14.79119 | **0.000001** | 12.17075 | **0.000002** | 13.27355 | 0.438347 |
| *V/H* | 72.0785 | 0.44951 | 73.27961 | 0.90215 | 72.10466 | 0.959646 | 73.12104 | 0.964124 | 70.58798 | 0.397256 | 72.29887 | 0.817802 |
| *κ* | 4.38779 | 0.6093 | 4.417335 | 0.737879 | 4.378019 | 0.319873 | 4.404082 | 0.670403 | 4.372562 | 0.449715 | 4.362559 | 0.988206 |
| *r* | 0.139053 | 0.4716 | 0.1406 | 0.913059 | 0.133691 | 0.723253 | 0.136509 | 0.999986 | 0.134572 | 0.929149 | 0.139217 | 0.794182 |
| *I_C_* | 1.46626 | **0** | 1.243785 | **0.044994** | 1.415078 | 0.162328 | 1.481767 | 0.589313 | 1.423657 | 0.721536 | 1.448869 | 0.978653 |
| *S_C_* | 0.087253 | **0.0001** | 0.083215 | 0.614346 | 0.088224 | 0.374146 | 0.080473 | 0.491355 | 0.089339 | 0.412069 | 0.084899 | 0.907877 |
| Study site: the PNG village Albulum | | | | | | | | | | | | |
|  | Baseline | | Intervention Year 1 | | Intervention Year 2 | | Intervention Year 3 | | Intervention Year 4 | | Intervention Year 5 | |
| Parameter  symbols | Median | KS test  *p*-value | Median | KS test  *p*-value | Median | KS test  *p*-value | Median | KS test  *p*-value | Median | KS test  *p*-value | Median | KS test  *p*-value |
| *λ* | 9.86941 | 0.5172 | 9.975978 | 0.999426 | 9.420708 | 0.910409 | 9.644291 | 0.999264 | 9.661415 | 0.937503 | 10.19795 | 0.922444 |
| *α* | 0.604992 | **0** | 0.619964 | 0.455232 | 0.58279 | 0.488846 | 0.608096 | 1 | 0.608382 | 0.9923 | 0.539825 | 0.42278 |
| *k_0_* | 0.0003 | 0.4056 | 0.000301 | 0.900951 | 0.000302 | 0.972436 | 0.000302 | 0.999993 | 0.000297 | 0.351459 | 0.0003 | 0.847408 |
| *k_Lin_* | 0.01787 | **0** | 0.014829 | 0.590743 | 0.017085 | 0.877733 | 0.022223 | 0.353495 | 0.0361 | **0.005277** | 0.027075 | 0.391056 |
| *σ* | 5.18246 | 0.1101 | 5.12158 | 0.999995 | 5.17799 | 0.999964 | 5.173517 | 0.982516 | 5.453849 | 0.241201 | 5.444814 | 0.65151 |
| *Ψ_1_* | 0.413249 | 0.1784 | 0.424014 | 0.974767 | 0.411207 | 0.987854 | 0.383332 | 0.388088 | 0.371257 | 0.946434 | 0.397656 | 0.609055 |
| *Ψ_2_s_2_* | 0.001652 | **0** | 0.001756 | 0.433523 | 0.001769 | 0.99994 | 0.001597 | 0.471415 | 0.001289 | 0.056907 | 0.001415 | 0.7146 |
| *μ* | 0.012941 | 0.151 | 0.012552 | 0.554057 | 0.012912 | 0.601418 | 0.012531 | 0.793592 | 0.012485 | 0.963829 | 0.012741 | 0.996679 |
| *γ* | 0.103089 | **0.0005** | 0.102922 | 0.999187 | 0.103138 | 0.999407 | 0.103089 | 0.998857 | 0.103355 | 0.999999 | 0.103462 | 0.984937 |
| *g* | 0.366707 | 0.6454 | 0.370308 | 0.999918 | 0.36568 | 0.99514 | 0.363013 | 0.999931 | 0.360363 | 0.999999 | 0.369021 | 0.953706 |
| *c* | 0.00797 | **0** | 0.008128 | 0.97638 | 0.00799 | 0.937863 | 0.008053 | 0.996727 | 0.007975 | 0.99998 | 0.008137 | 0.99829 |
| *δ* | 0.0 | 0.999999 | 0.0 | 0.999997 | 0.0 | 0.999997 | 0.0 | 0.999997 | 0.0 | 0.999997 | 0.0 | 0.999997 |
| *H_lin_* | 12.4467 | **0** | 14.90763 | **0.000029** | 13.11034 | **0.0151** | 14.76481 | **0.035829** | 15.74912 | 0.140235 | 13.48292 | **0.002066** |
| *V/H* | 357.401 | 0.5172 | 353.5848 | 0.999426 | 374.429 | 0.910409 | 365.7431 | 0.999264 | 365.0955 | 0.937503 | 345.8865 | 0.922444 |
| *κ* | 4.37356 | 0.4845 | 4.350934 | 0.999984 | 4.395675 | 0.939901 | 4.340003 | 0.560084 | 4.342036 | 0.999989 | 4.373918 | 0.745948 |
| *r* | 0.13463 | 0.2132 | 0.140023 | 0.960476 | 0.135012 | 0.996949 | 0.13463 | 0.997925 | 0.125517 | 0.759787 | 0.131422 | 0.958705 |
| *I_C_* | 1.84285 | **0** | 1.564488 | 0.106218 | 1.752043 | 0.601418 | 1.687087 | 0.973889 | 1.724599 | 0.99091 | 1.780527 | 0.987797 |
| *S_C_* | 0.095863 | **0.00882** | 0.09503 | 0.99992 | 0.095872 | 0.999957 | 0.094861 | 0.999998 | 0.08179 | 0.416403 | 0.096343 | 0.325763 |
| Study site: the PNG village Yauatong | | | | | | | | | | | | |
|  | Baseline | | Intervention Year 1 | | Intervention Year 2 | | Intervention Year 3 | | Intervention Year 4 | | Intervention Year 5 | |
| Parameter  symbols | Median | KS test  *p*-value | Median | KS test  *p*-value | Median | KS test  *p*-value | Median | KS test  *p*-value | Median | KS test  *p*-value | Median | KS test  *p*-value |
| *λ* | 9.98429 | 0.869 | 9.9491 | 0.968235 | 10.05889 | 0.999958 | 9.977146 | 0.99887 | 10.10628 | 0.999997 | 9.831391 | 0.697755 |
| *α* | 0.6997 | **0** | 0.6991 | 0.9965 | 0.675626 | 0.888175 | 0.715999 | 0.941765 | 0.687881 | 0.991259 | 0.612853 | 0.432666 |
| *k_0_* | 0.000499 | 0.8162 | 0.0005 | 0.503101 | 0.000497 | 0.811349 | 0.000499 | 0.921919 | 0.000497 | 0.975168 | 0.0005 | 0.522227 |
| *k_Lin_* | 0.018227 | **0** | 0.0188 | 0.948038 | 0.020707 | 0.849704 | 0.02681 | 0.528375 | 0.028921 | 0.999921 | 0.028294 | 0.801953 |
| *σ* | 5.16192 | 0.4205 | 5.4221 | 0.943975 | 5.42205 | 0.999978 | 5.78323 | 0.711959 | 5.730321 | 1 | 5.286687 | 0.627752 |
| *Ψ_1_* | 0.434446 | 0.7227 | 0.4602 | 0.924812 | 0.410736 | 0.985954 | 0.39807 | 0.97824 | 0.41657 | 0.994302 | 0.388443 | 0.660187 |
| *Ψ_2_s_2_* | 0.001975 | **0.002253** | 0.0021 | 0.983442 | 0.001966 | 0.895422 | 0.001479 | 0.157613 | 0.001651 | 0.999217 | 0.001853 | 0.578955 |
| *μ* | 0.014232 | **0** | 0.0138 | 0.621659 | 0.012875 | 0.698064 | 0.013686 | 0.965339 | 0.014252 | 0.999891 | 0.014438 | 0.807608 |
| *γ* | 0.103568 | **0.0002** | 0.1036 | 0.967161 | 0.103161 | 0.999587 | 0.104574 | 0.90494 | 0.099231 | 0.609333 | 0.103939 | 0.522227 |
| *g* | 0.361163 | 0.0828 | 0.3692 | 0.540616 | 0.361627 | 0.945641 | 0.356224 | 0.999786 | 0.355595 | 1 | 0.356885 | 0.994396 |
| *c* | 0.007639 | **0** | 0.0075 | 0.919817 | 0.007743 | 0.912489 | 0.007138 | 0.705322 | 0.007353 | 1 | 0.007102 | 0.998435 |
| *δ* | 0.0 | 0.999999 | 0.0 | 0.999997 | 0.0 | 0.999997 | 0.0 | 0.999997 | 0.0 | 0.999997 | 0.0 | 0.999997 |
| *H_lin_* | 12.4222 | **0** | 17.929 | **0** | 18.30862 | 0.664905 | 18.6313 | 0.74045 | 18.06581 | 0.798388 | 16.47265 | **0.007564** |
| *V/H* | 309.252 | 0.869 | 310.35 | 0.968235 | 306.959 | 0.999958 | 309.4947 | 0.99887 | 305.5197 | 0.999997 | 314.0681 | 0.697755 |
| *κ* | 4.40209 | 0.6837 | 4.414 | 0.822883 | 4.451416 | 0.868078 | 4.543682 | 0.560772 | 4.512196 | 0.994302 | 4.398383 | 0.442704 |
| *r* | 0.142207 | 0.3045 | 0.1422 | 0.859406 | 0.14982 | 0.840154 | 0.133595 | 0.90494 | 0.128982 | 1 | 0.14315 | 0.893036 |
| *I_C_* | 1.08396 | **0** | 1.007 | 0.838522 | 1.072629 | 0.999999 | 1.077788 | 0.996664 | 1.073444 | 0.999999 | 1.10081 | 0.987045 |
| *S_C_* | 0.092925 | **0.0113** | 0.08 | 0.831095 | 0.079991 | 1 | 0.068386 | 0.716373 | 0.07193 | 0.939068 | 0.086621 | 0.665418 |
| Study site: the PNG village Nanaha | | | | | | | | | | | | |
|  | Baseline | | Intervention Year 1 | | Intervention Year 2 | | Intervention Year 3 | | Intervention Year 4 | | Intervention Year 5 | |
| Parameter  symbols | Median | KS test  *p*-value | Median | KS test  *p*-value | Median | KS test  *p*-value | Median | KS test  *p*-value | Median | KS test  *p*-value | Median | KS test  *p*-value |
| *λ* | 9.69295 | **0.004481** | 9.411051 | 0.850917 | 9.724008 | 0.691534 | 9.486115 | 1 | 9.570489 | 1 | 9.485868 | 1 |
| *α* | 0.735955 | **0.0015** | 0.841992 | 0.934405 | 0.736676 | 0.942084 | 0.735955 | 1 | 0.707295 | 0.999935 | 0.711832 | 1 |
| *k_0_* | 0.000296 | **0.002409** | 0.000296 | 0.999753 | 0.000296 | 0.999747 | 0.000294 | 0.999346 | 0.000294 | 1 | 0.000296 | 0.999896 |
| *k_Lin_* | 0.001456 | **0** | 0.00165 | 0.995237 | 0.001447 | 0.990773 | 0.001436 | 1 | 0.001493 | 0.999963 | 0.001456 | 1 |
| *σ* | 5.42712 | **0.001274** | 5.61398 | 0.990502 | 5.358813 | 0.999346 | 5.679844 | 0.999994 | 5.61398 | 1 | 5.409244 | 0.999678 |
| *Ψ_1_* | 0.39075 | **0.002081** | 0.375731 | 0.975927 | 0.384601 | 0.985336 | 0.387253 | 1 | 0.367575 | 0.972798 | 0.381315 | 1 |
| *Ψ_2_s_2_* | 0.001537 | **0.0003** | 0.001214 | 0.38574 | 0.0015 | 0.539201 | 0.001588 | 0.997931 | 0.001549 | 1 | 0.001528 | 1 |
| *μ* | 0.014059 | **0.0016** | 0.01302 | 0.368538 | 0.013906 | 0.599407 | 0.014038 | 1 | 0.014043 | 1 | 0.014038 | 1 |
| *γ* | 0.09821 | **0.0043** | 0.101855 | 0.947724 | 0.097732 | 0.861247 | 0.099239 | 0.999864 | 0.097223 | 0.998295 | 0.097253 | 1 |
| *g* | 0.366025 | **0.006099** | 0.379726 | 0.93191 | 0.366433 | 0.968378 | 0.366022 | 1 | 0.359234 | 1 | 0.362916 | 0.993086 |
| *c* | 4.90E-06 | **0.002713** | 0.000005 | 0.999996 | 0.000005 | 1 | 0.000005 | 1 | 0.000005 | 0.998003 | 0.000005 | 1 |
| *δ* | 0.0 | 0.999999 | 0.0 | 0.999997 | 0.0 | 0.999997 | 0.0 | 0.999997 | 0.0 | 0.999997 | 0.0 | 0.999997 |
| *H_lin_* | 4.07813 | **0** | 3.546625 | 0.747787 | 4.005658 | 0.691534 | 4.288231 | 0.921244 | 4.160852 | 1 | 4.141356 | 1 |
| *V/H* | 99.8234 | 0.4481 | 102.8201 | 0.850917 | 99.50548 | 0.691534 | 102 | 1 | 101.1086 | 1 | 102.0026 | 1 |
| *κ* | 4.3065 | **0.00636** | 4.25819 | 0.987456 | 4.306175 | 0.990773 | 4.306497 | 1 | 4.331718 | 1 | 4.307605 | 1 |
| *r* | 0.14532 | **0.004471** | 0.148002 | 0.811146 | 0.145546 | 0.781037 | 0.144809 | 1 | 0.141828 | 1 | 0.145771 | 0.999932 |
| *I_C_* | 0.867188 | **0** | 0.803345 | 0.99283 | 0.864034 | 0.994562 | 0.906478 | 0.988501 | 0.911719 | 1 | 0.906478 | 1 |
| *S_C_* | 0.11486 | **0.001072** | 0.122799 | 0.996514 | 0.114746 | 0.985336 | 0.116836 | 0.99928 | 0.113506 | 0.998684 | 0.112531 | 0.999991 |
| Study site: the PNG village Ngahmbule | | | | | | | | | | | | |
|  | Baseline | | Intervention Year 1 | | Intervention Year 2 | | Intervention Year 3 | | Intervention Year 4 | | Intervention Year 5 | |
| Parameter  symbols | Median | KS test  *p*-value | Median | KS test  *p*-value | Median | KS test  *p*-value | Median | KS test  *p*-value | Median | KS test  *p*-value | Median | KS test  *p*-value |
| *λ* | 10.1956 | **0.009285** | 10.417 | 1 | 10.31189 | 1 | 10.15214 | 1 | 9.984423 | 1 | 10.20694 | 1 |
| *α* | 0.787931 | **0.0005** | 0.8044 | 0.999432 | 0.786932 | 0.999812 | 0.785792 | 1 | 0.786932 | 1 | 0.799586 | 0.999985 |
| *k_0_* | 0.000705 | **0.002637** | 0.0007 | 1 | 0.000705 | 1 | 0.000705 | 1 | 0.000705 | 1 | 0.000701 | 0.953707 |
| *k_Lin_* | 0.005352 | **0** | 0.0052 | 1 | 0.005419 | 1 | 0.005554 | 1 | 0.005626 | 1 | 0.005581 | 1 |
| *σ* | 4.98153 | **0.002556** | 5.1638 | 0.999998 | 4.807569 | 0.979496 | 5.079597 | 0.999971 | 4.995047 | 1 | 4.981529 | 0.999999 |
| *Ψ_1_* | 0.407579 | **0.001797** | 0.4008 | 1 | 0.400831 | 1 | 0.404591 | 0.999998 | 0.413426 | 1 | 0.39777 | 0.999999 |
| *Ψ_2_s_2_* | 0.001897 | **0.0094** | 0.0019 | 1 | 0.00189 | 1 | 0.001854 | 0.999997 | 0.001979 | 0.999987 | 0.001839 | 0.999896 |
| *μ* | 0.01201 | **0.001322** | 0.012 | 0.983792 | 0.01203 | 0.960003 | 0.011993 | 1 | 0.01218 | 0.999987 | 0.012221 | 1 |
| *γ* | 0.102749 | **0.00627** | 0.1011 | 0.998457 | 0.102716 | 0.999776 | 0.101934 | 1 | 0.102716 | 1 | 0.102549 | 1 |
| *g* | 0.376026 | **0.008992** | 0.3794 | 1 | 0.375101 | 1 | 0.371703 | 1 | 0.369696 | 0.999987 | 0.379185 | 0.999922 |
| *c* | 5.64E-06 | **0.002073** | 5E-06 | 1 | 0.000006 | 1 | 0.000005 | 1 | 0.000006 | 0.999987 | 0.000006 | 0.999998 |
| *δ* | 0.0 | 0.999999 | 0.0 | 0.999997 | 0.0 | 0.999997 | 0.0 | 0.999997 | 0.0 | 0.999997 | 0.0 | 0.999997 |
| *H_lin_* | 15.2962 | **0** | 16.177 | 0.230503 | 15.57061 | 0.575581 | 15.57061 | 1 | 15.12164 | 0.993629 | 15.2962 | 0.999842 |
| *V/H* | 35.522 | 0.9285 | 34.767 | 1 | 35.12219 | 1 | 35.67458 | 1 | 36.27362 | 1 | 35.4824 | 1 |
| *κ* | 4.39846 | **0.002049** | 4.405 | 0.999719 | 4.400739 | 0.999998 | 4.404955 | 1 | 4.388846 | 1 | 4.40689 | 1 |
| *r* | 0.162137 | **0** | 0.1607 | 0.995315 | 0.163741 | 0.974276 | 0.162125 | 1 | 0.162125 | 1 | 0.162137 | 1 |
| *I_C_* | 0.867555 | **0** | 0.868 | 1 | 0.86577 | 1 | 0.868039 | 1 | 0.841086 | 0.999987 | 0.868523 | 0.999794 |
| *S_C_* | 0.107619 | **0.004659** | 0.1061 | 1 | 0.105776 | 1 | 0.104748 | 1 | 0.106265 | 1 | 0.107994 | 1 |

**Table S3 -** **Results of the univariate Kolmogorov-Smirnov (KS) test of differences between prior and posterior distributions of drug related parameters.** The *p*-value shows the significance level of the KS test. A *p*-value <0.05 indicates that posterior distribution is significantly different from its prior, which is always the case for the worm killing efficacy parameter.

|  | Intervention Year 1 | | Intervention Year 2 | | Intervention Year 3 | | Intervention Year 4 | | Intervention Year 5 | |
| --- | --- | --- | --- | --- | --- | --- | --- | --- | --- | --- |
| Peneng | Median | KS test  *p*-value | Median | KS test  *p*-value | Median | KS test  *p*-value | Median | KS test  *p*-value | Median | KS test  *p*-value |
| Worm killing efficacy | 38.05 | 0 | 72.40 | 0 | 62.86 | 0 | 73.84 | 0 | 69.66 | 0 |
| Mf killing efficacy | 73.98 | 0.178204 | 78.63 | 0.000008 | 75.24 | 0.840727 | 74.74 | 0.219441 | 73.78 | 0.309879 |
| Worm fecundity reduction efficacy | 76.39 | 0.271467 | 75.69 | 0.600274 | 76.24 | 0.065465 | 74.72 | 0.890737 | 73.83 | 0.54142 |
| Waning period (months) | 3 | 0.213686 | 4 | 0.137348 | 4 | 0.801864 | 4 | 0.405727 | 4 | 0.754289 |
| Albulum |  |  |  |  |  |  |  |  |  |  |
| Worm killing efficacy | 46.48 | 0.121075 | 73.95 | 0 | 70.79 | 0 | 67.88 | 0 | 77.37 | 0 |
| Mf killing efficacy | 73.45 | 0.035724 | 79.16 | 0.000001 | 77.14 | 0.026579 | 76.82 | 0.015931 | 79.85 | 0 |
| Worm fecundity reduction efficacy | 73.89 | 0.278237 | 76.76 | 0.153407 | 76.40 | 0.199462 | 76.14 | 0.0043 | 76.16 | 0.197992 |
| Waning period (months) | 3 | 0.063861 | 4 | 0.003456 | 4 | 0.06687 | 4 | 0.298866 | 4 | 0.00001 |
| Yauatong |  |  |  |  |  |  |  |  |  |  |
| Worm killing efficacy | 68.54 | 0 | 73.88 | 0 | 69.71 | 0 | 70.11 | 0 | 79.99 | 0 |
| Mf killing efficacy | 79.38 | 0.000004 | 78.55 | 0.000329 | 76.55 | 0.095626 | 79.04 | 0.001428 | 80.32 | 0 |
| Worm fecundity reduction efficacy | 73.75 | 0.242557 | 73.93 | 0.732421 | 73.97 | 0.336959 | 75.02 | 0.969358 | 74.55 | 0.943816 |
| Waning period (months) | 4 | 0.009211 | 4 | 0.000147 | 4 | 0.892152 | 4 | 0.040003 | 4 | 0 |
| Nanaha |  |  |  |  |  |  |  |  |  |  |
| Worm killing efficacy | 21.95 | 0 | 45.03 | 0 | 60.95 | 0 | 81.45 | 0 | 80.42 | 0 |
| Mf killing efficacy | 70.13 | 0 | 74.26 | 0.500176 | 74.99 | 0.977677 | 83.32 | 0 | 80.26 | 0 |
| Worm fecundity reduction efficacy | 72.83 | 0.078796 | 75.17 | 0.852811 | 76.55 | 0.117248 | 78.87 | 0.000048 | 76.73 | 0.067844 |
| Waning period (months) | 3 | 0 | 3 | 0.311486 | 4 | 0.33216 | 4 | 0 | 4 | 0 |
| Ngahmbule |  |  |  |  |  |  |  |  |  |  |
| Worm killing efficacy | 51.63 | 0.00004 | 50.07 | 0 | 74.47 | 0 | 78.32 | 0 | 80.94 | 0 |
| Mf killing efficacy | 75.02 | 0.670438 | 72.42 | 0.015551 | 75.27 | 0.579461 | 79.57 | 0 | 81.38 | 0 |
| Worm fecundity reduction efficacy | 76.22 | 0.528885 | 74.66 | 0.95527 | 76.29 | 0.762766 | 75.13 | 0.939772 | 76.82 | 0.20995 |
| Waning period (months) | 4 | 0.791896 | 3 | 0.971471 | 4 | 0.000396 | 4 | 0.000001 | 4 | 0.000159 |

**Table S4 - Monte Carlo *p*-values for the backfitted models to the five baseline data**

| Age-group:--> | 0 - 10 | 10 - 20 | 20 - 30 | 30 - 40 | 40 - 50 | 50 - 60 | 60 - 70 | 70 - 80 | Overall |
| --- | --- | --- | --- | --- | --- | --- | --- | --- | --- |
|  |  |  | Monte Carlo *p*-values for Albulum | | | |  |  |  |
| * | 0.751 | 0.985 | 0.247 | 0.814 | 0.992 | 0.873 | 0.999 | N/A | 0.998 |
| 1 | 0.847 | 0.998 | 0.238 | 0.403 | 0.835 | 0.968 | 0.999 | N/A | 0.999 |
| 2 | 0.882 | 0.999 | 0.279 | 0.491 | 0.882 | 0.98 | 0.999 | N/A | 0.999 |
| 3 | 0.84 | 0.996 | 0.182 | 0.476 | 0.846 | 0.964 | 0.999 | N/A | 0.999 |
| 4 | 0.823 | 0.998 | 0.218 | 0.466 | 0.863 | 0.952 | 0.999 | N/A | 0.999 |
| 5 | 0.83 | 0.999 | 0.276 | 0.428 | 0.86 | 0.976 | 0.999 | N/A | 0.999 |
|  |  |  | Monte Carlo *p*-values for Yauatong | | | |  |  |  |
| * | 0.793 | 0.999 | 0.878 | 0.99 | 0.997 | 0.999 | 0.054 | N/A | 0.997 |
| 1 | 0.999 | 0.556 | 0.265 | 0.967 | 0.995 | 0.999 | 0.444 | N/A | 0.986 |
| 2 | 0.999 | 0.544 | 0.294 | 0.973 | 0.999 | 0.999 | 0.34 | N/A | 0.988 |
| 3 | 0.999 | 0.9 | 0.879 | 0.958 | 0.977 | 0.993 | 0.268 | N/A | 0.951 |
| 4 | 0.999 | 0.634 | 0.301 | 0.961 | 0.999 | 0.999 | 0.338 | N/A | 0.983 |
| 5 | 0.999 | 0.465 | 0.15 | 0.974 | 0.998 | 0.999 | 0.479 | N/A | 0.992 |
|  |  |  | Monte Carlo *p*-values for Nanaha | | | |  |  |  |
| * | 0.993 | 0.964 | 0.754 | 0.312 | 0.681 | 0.996 | 0.999 | 0.999 | 0.99 |
| 1 | 0.956 | 0.998 | 0.999 | 0.99 | 0.999 | 0.709 | 0.701 | 0.985 | 0.998 |
| 2 | 0.9 | 0.999 | 0.999 | 0.99 | 0.999 | 0.689 | 0.671 | 0.978 | 0.996 |
| 3 | 0.87 | 0.999 | 0.999 | 0.99 | 0.999 | 0.677 | 0.665 | 0.99 | 0.999 |
| 4 | 0.864 | 0.999 | 0.999 | 0.989 | 0.999 | 0.54 | 0.533 | 0.994 | 0.999 |
| 5 | 0.881 | 0.999 | 0.999 | 0.99 | 0.999 | 0.698 | 0.675 | 0.982 | 0.999 |
|  |  |  | Monte Carlo *p*-values for Ngahmbule | | | |  |  |  |
| * | 0.957 | 0.73 | 0.823 | 0.553 | 0.504 | 0.986 | 0.872 | 0.674 | 0.998 |
| 1 | 0.98 | 0.503 | 0.613 | 0.151 | 0.445 | 0.683 | 0.451 | 0.584 | 0.933 |
| 2 | 0.976 | 0.494 | 0.588 | 0.232 | 0.412 | 0.716 | 0.56 | 0.566 | 0.948 |
| 3 | 0.964 | 0.538 | 0.6 | 0.154 | 0.356 | 0.71 | 0.404 | 0.51 | 0.93 |
| 4 | 0.978 | 0.572 | 0.632 | 0.184 | 0.472 | 0.658 | 0.426 | 0.59 | 0.934 |
| 5 | 0.974 | 0.603 | 0.601 | 0.175 | 0.452 | 0.662 | 0.448 | 0.572 | 0.953 |

(*) The *p*-values are calculated for the model fits directly fitted to the baseline data. The numbers in the first column represent the intervention years. In this case, the posteriors of the non-drug parameters obtained from the sequential model fits to those years’ infection data were used to reconstruct the baseline mf age-prevalence curves in the right panel of **Figures** **S5** to **S8**. N/A represents that there were either no observed data in the age-group of the study village in question during their baseline survey.
